# Temperature and injection water source influence microbial community structure in four Alaskan North Slope hydrocarbon reservoirs

**DOI:** 10.3389/fmicb.2014.00409

**Published:** 2014-08-07

**Authors:** Yvette M. Piceno, Francine C. Reid, Lauren M. Tom, Mark E. Conrad, Markus Bill, Christopher G. Hubbard, Bruce W. Fouke, Craig J. Graff, Jiabin Han, William T. Stringfellow, Jeremy S. Hanlon, Ping Hu, Terry C. Hazen, Gary L. Andersen

**Affiliations:** ^1^Earth Sciences Division, Lawrence Berkeley National LaboratoryBerkeley, CA, USA; ^2^Energy Biosciences InstituteBerkeley, CA, USA; ^3^Department of Geology, University of Illinois at Urbana-Champaign, Urbana-ChampaignIL, USA; ^4^Production Chemistry, BP ExplorationAnchorage, AK, USA; ^5^Ecological Engineering Research Program, University of the PacificStockton, CA, USA; ^6^Department of Civil and Environmental Engineering, University of TennesseeKnoxville, TN, USA

**Keywords:** petroleum, reservoir, microbiology, phylochip, stable isotopes

## Abstract

A fundamental knowledge of microbial community structure in petroleum reservoirs can improve predictive modeling of these environments. We used hydrocarbon profiles, stable isotopes, and high-density DNA microarray analysis to characterize microbial communities in produced water from four Alaskan North Slope hydrocarbon reservoirs. Produced fluids from Schrader Bluff (24–27°C), Kuparuk (47–70°C), Sag River (80°C), and Ivishak (80–83°C) reservoirs were collected, with paired soured/non-soured wells sampled from Kuparuk and Ivishak. Chemical and stable isotope data suggested Schrader Bluff had substantial biogenic methane, whereas methane was mostly thermogenic in deeper reservoirs. Acetoclastic methanogens (*Methanosaeta*) were most prominent in Schrader Bluff samples, and the combined δD and δ^13^C values of methane also indicated acetoclastic methanogenesis could be a primary route for biogenic methane. Conversely, hydrogenotrophic methanogens (e.g., Methanobacteriaceae) and sulfide-producing *Archaeoglobus* and *Thermococcus* were more prominent in Kuparuk samples. Sulfide-producing microbes were detected in all reservoirs, uncoupled from souring status (e.g., the non-soured Kuparuk samples had higher relative abundances of many sulfate-reducers compared to the soured sample, suggesting sulfate-reducers may be living fermentatively/syntrophically when sulfate is limited). Sulfate abundance via long-term seawater injection resulted in greater relative abundances of *Desulfonauticus*, *Desulfomicrobium*, and *Desulfuromonas* in the soured Ivishak well compared to the non-soured well. In the non-soured Ivishak sample, several taxa affiliated with *Thermoanaerobacter* and *Halomonas* predominated. Archaea were not detected in the deepest reservoirs. Functional group taxa differed in relative abundance among reservoirs, likely reflecting differing thermal and/or geochemical influences.

## Introduction

Oil reservoir hydrogen sulfide production (commonly referred to as souring) poses health risks to workers and is estimated to cost the petroleum industry billions of dollars per year (Chen et al., 1994) due to lowered hydrocarbon quality and increased infrastructure maintenance related to corrosion (Gieg et al., 2011). Sulfide-producing microorganisms (SPM) are responsible for much of the sulfides in produced oil and gas fluids, especially after water injection has begun to enhance oil recovery, yet not all reservoirs undergoing secondary recovery produce measurable sulfides. Because of the complexities of reservoir geohydromechanics and both limitations and costs associated with collecting samples from deep subsurface oil reservoirs, it is challenging to predict when a reservoir will start to sour. To increase the predictiveness of the process, models need to be parameterized, including information about existing microbial communities.

Oil reservoirs at or below roughly 80°C commonly show signs of hydrocarbon biodegradation (Wilhelms et al., 2001; Head et al., 2003, 2010; Aitken et al., 2004; Huang and Larter, 2005). Deep oil reservoirs are anaerobic, and biodegradation commonly proceeds through to methanogenesis (Jones et al., 2008; Meslé et al., 2013) or, if more thermodynamically favorable electron acceptors are available, to respiratory pathways such as sulfate-reduction (Head et al., 2010; Callbeck et al., 2013). Fermentation processes linked to hydrocarbon degradation provide substrates such as organic acids, alcohols, H_2_, and CO_2_ that various microbes can use. Additionally, nutrients and electron acceptors provided by formation waters (Head et al., 2003) and/or injection waters can sustain or potentially stimulate biodegradation and especially souring (Mueller and Nielsen, 1996; Gieg et al., 2011). Why some wells sour, while others do not, remains a question of great economic consequence. Several studies have employed culture-based and culture-independent methods to define microbial communities from produced fluids from oil reservoirs, including separate analysis of water, oil, and solids from produced fluids (Kobayashi et al., 2012; Wang et al., 2014), providing insights about microbes residing in oil reservoirs and processes contributing to souring (Mueller and Nielsen, 1996; Nilsen et al., 1996a,b; Orphan et al., 2000; Jeanthon et al., 2005; Magot, 2005; Ollivier and Cayol, 2005; Rabus, 2005; Kleinsteuber et al., 2012; Hasegawa et al., 2014; Okoro et al., 2014).

Alaska's North Slope has been the focus of several studies aimed at understanding souring and related corrosion processes (Stetter et al., 1993; Chen et al., 1994; Mueller and Nielsen, 1996; Duncan et al., 2009; Pham et al., 2009; Gieg et al., 2010). Recent studies have used sequencing of fosmids and/or sequencing of PCR-amplified 16S rRNA genes to examine produced water or enrichments from two oil reservoirs in the region (Duncan et al., 2009; Pham et al., 2009; Gieg et al., 2010). PhyloChip microarray analysis provides taxonomic and relative abundance information about bacterial and archaeal constituents of microbial communities spanning several orders of magnitude in abundance (Probst et al., 2014) and has been used to assess microbial community structure differences among samples from many environments (Brodie et al., 2006, 2007; DeSantis et al., 2007; Hazen et al., 2010). For the current study, we examined produced water and oil samples from four Alaskan oil reservoirs that varied in temperature (from 24 to 83°C) and injection water history. We present 16S rRNA gene-based microarray data from these mesothermic and hyperthermic reservoirs, supplemented with isotopic and geochemical data. The primary goals were to describe differences in the microbial communities of oil reservoirs varying in temperature and injection water history and to assess differences between soured and non-soured wells.

## Materials and methods

### Geological setting

The Milne Point and adjacent Prudhoe Bay fields (parcels of land) are located on the North Slope coastal plain of Alaska within the hydrocarbon-rich Arctic Petroleum Province (see maps in Kornbrath et al., 1997; ANWR, 1998; Houseknecht and Bird, 2005; Bird and Houseknecht, 2011). These fields lie between the National Petroleum Reserve of Alaska (NPRA) and the Arctic National Wildlife Refuge (ANWR, 1998; BP, 2013). The Milne Point field has already produced more than 0.3 billion barrels (gross) and has at least 8.6 billion barrels (gross) of remaining conventional and non-conventional oil in place (Houseknecht and Bird, 2005; Bird and Houseknecht, 2011; BP, 2013). The regional geological history of the North Slope is comprised of four stratigraphic sequences, each of which reflects large-scale tectonic cycles and associated significant changes in depositional environment and structural framework (ANWR, 1998; Houseknecht and Bird, 2005). These include (Figure 2 in Houseknecht and Bird, 2005): (1) the Franklinian sequence (Late Proterozoic economic basement through Devonian); (2) the Ellesmerian sequence (Mississippian through Triassic passive margin deposition); (3) the Beaufortian sequence (Jurassic through Lower Cretaceous synrift deposition); and (4) the Brookian sequence (Cretaceous through Tertiary foreland basin and passive margin deposition). Mixed oil-water-gas samples were collected for this study from Milne Point and Prudhoe Bay production wells that penetrate the Ellesmerian sequence (Ivishak and Sag River Formations) and the Beaufortian sequence (Kuparuk Formation), which produce lighter crude oil. Samples were also collected from the Brookian sequence (Schrader Bluff Formation), which produces viscous crude oil (BP, 2013). Hydrocarbons within the Milne Point field share a complex history with those of neighboring fields that involved multiple sources, up-section migration, evaporative fractionation, and microbial biodegradation (Masterson et al., 2001).

A brief overview of the sedimentology and stratigraphy of the subsurface reservoirs of the fields sampled in this study are as follows (physical conditions summarized in Table 1 and stratigraphy depicted in Figure S1; also see stratigraphic sections in Masterson et al., 2001; Houseknecht and Bird, 2005; Kulander et al., 2005). Information about a fifth formation that provides make-up injection water for two of the formations also is presented. Formation temperatures were calculated based on depth [1.11°C/30.48 m (2°F/100 ft)]. The Ivishak Formation is Early Triassic in age and composed of off-lapping fluvially-dominated deltaic wedges of marginal marine siliciclastic sediment deposits (Figure 2 in Tye et al., 1999). The Ivishak is buried at nearly 9000 ft (2743 m) total vertical depth subsea (TVDSS) (i.e., relative to mean sealevel) and currently has a subsurface temperature (T) of 80–83°C, with temperatures as great as 110°C in places when first assessed (Masterson et al., 2001). The reservoir portion of the Ivishak Formation contains complex associations of distributary mouth bar, channel, and fluvial facies deposits that include coarse sandstone, shale, and silt lithologies (Tye et al., 1999). The Sag River Formation (TVDSS ≥ 8500 ft [2590 m]; *T* = 80°C) is Late Triassic to Early Jurassic in depositional age, is relatively thin (<40 m-thick), and is composed of coarse-grained marine shelf siliciclastic sandstone that interfingers with the Shublik Formation (Barnes, 2001; Kulander et al., 2005). The Kuparuk Formation (TVDSS ≥ 5900–7000 ft [1800–2133 m]; *T* = 47 to 70°C) is an Early Cretaceous stratigraphic sequence of transgressive-lag marine shelf siliciclastic sandstone deposits (Carman and Hardwick, 1983; Masterson and Eggert, 1992). The Schrader Bluff (TVDSS ≥ 3700 ft [1128 m]; *T* = 24 to 27°C) and Prince Creek Formations (TVDSS ≥ 2000 ft [610 m]; *T* = 8°C) were deposited in laterally inter-tonguing marginal marine depositional environments during the Late Cretaceous (Flores et al., 2007). The contact between these formations is highly transgressive in nature and portions of the Prince Creek may be as young as Paleocene (Decker, 2007). The Schrader Bluff is primarily composed of coarse shallow marine shelf siliciclastic sandstone, while the Prince Creek is comprised of coarse fluvial siliciclastic sandstone. The Prince Creek Formation was syndepositonally deposited with the Schrader Bluff Formation.

**Table 1:** **Formation physical characteristics, produced water lift method, and fluid mixing for Milne Point and Prudhoe Bay (Alaskan North Slope) oil field samples**.

**Formation/Reservoir**	**Depth (m)^c^**	**Temperature (°C)^d^**	**Well**	**Injection influence in the reservoir^e^**	**Artifical lift method**	**Fluid mixed in wellbore**
Prince Creek^a^	700–760	7.8	PC	None	ESP	None
Schrader Bluff^a^	1200–1400	24–27	SB1	Mixed Water (Kuparuk, Schrader Bluff, Sag River, Ugnu, Prince Creek)	ESP	None
Schrader Bluff^a^			SB2	Mixed Water (Kuparuk, Schrader Bluff, Sag River, Ugnu, Prince Creek)	ESP	None
Kuparuk River^a^	1785–2150	47–70	K1	Mixed Water (Kuparuk, Schrader Bluff, Sag River, Ugnu, Prince Creek)	ESP	None
Kuparuk River^a^			K2	Prince Creek	ESP	None
Kuparuk River^b^			K3	Mixed Water (Ivishak, Kuparuk, Schrader Bluff)	Gas lift	Natural gas
Sag River^a^	2690–2730	80	SR1	None	Jet pump	PC water
Ivishak^b^	2750–3100	80-83	I1	Seawater	Gas lift	Natural gas
Ivishak^b^			I2	Seawater/Ivishak	Gas lift	Natural gas

a Milne Point field.

b Prudhoe Bay field.

c Approximate mean vertical depth subsea reported in the literature.

d Average formation temperatures were calculated based on formation depth.

e Water produced from a reservoir is often injected back into the same or another reservoir.

### Alaskan north slope production wells

Production from many Alaskan reservoirs is integrated. Produced fluids from the various reservoirs can mix due to water injection and/or artificial lift. As oil and gas is removed from the rock formations, water is injected at other locations to sweep more oil toward the producing well and to maintain the original reservoir pressure (as illustrated in Planckaert, 2005). The “sweep” (or flood) water can come from various sources and may change the reservoir chemistry, often cooling the reservoir for some distance around the injection well. The Prince Creek sample was taken from a formation used solely to source water for secondary production of oil-bearing formations. The other samples for this study (collected from wellheads) are representative of mixed fluids. Schrader Bluff wells and one Kuparuk well were influenced by injection of a mixed water consisting of Kuparuk, Schrader Bluff, Sag River, Ugnu (another formation), and Prince Creek produced water (Table 1). One Kuparuk well was influenced by a mixed water consisting of Ivishak, Kuparuk, and Schrader Bluff Formation water. The well sampled from Sag River did not require secondary production, but the sample was mixed with Prince Creek water used for artificial lift, as described below. One of the Ivishak wells drilled more recently was influenced solely by seawater injection, but seawater has yet to break-through. The other Ivishak well was influenced by seawater and Ivishak water injection over a period of many years and had seawater break-through.

Insufficient reservoir pressures in these four reservoirs required artificial lift to bring fluids to the surface. Production fluids from many of the wells were lifted with electric submersible pumps (ESP) placed at the bottom of a well (Table 1). These wells only produce fluids that have come from the reservoir. Three of the wells were lifted with gas. The fluids from these wells were mixed with high-pressure natural gas through several thousand feet of production tubing before reaching the sampling point at the surface. The Sag River produced fluid was lifted with a jet pump, which used Prince Creek Formation water as a power fluid. This jet pump mixed the Prince Creek water with the Sag River oil and gas for several thousand feet prior to reaching the sampling point at the surface.

### Sample collection and handling

**Figure 1:**
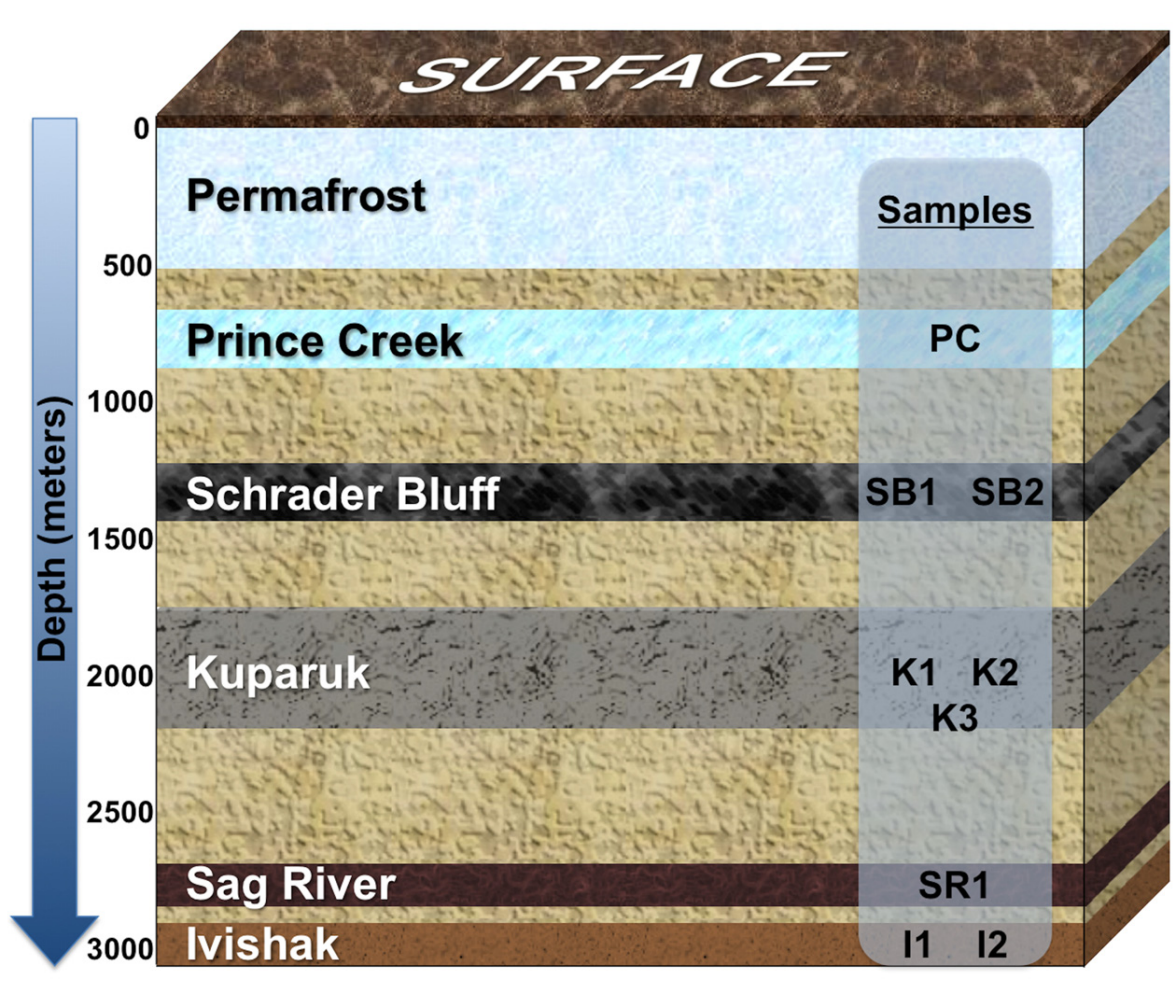
**Schematic showing relative positions of geological formations in the Milne Point and/or Prudhoe Bay fields, Alaskan North Slope**. Formations extend over many miles of the Milne Point and/or Prudhoe Bay fields and may tilt in regions, so depth range averages were used to depict the relative positions of the formations; the depth of each formation is not to scale. Prince Creek Formation water is used as make-up water supporting secondary recovery from Schrader Bluff and Kuparuk Formations. Prince Creek water is also used for artificial lift of Sag River produced fluids. Seawater injection is used to support secondary recovery from the Ivishak reservoir. Wells sampled for this study are labeled according to the formation from which they produce.

Milne Point and Prudhoe Bay oil field produced water samples were collected from eight wellheads in 2011 or 2013. Water used to support secondary recovery or artificial lift also was sampled in 2011. Samples for genomic analysis from 2011 were passed through 0.2 micron filters (Supor^®^ MicroFunnel, Pall Corp., Port Washington, NY) at a field lab and the filters were shipped to Lawrence Berkeley National Laboratory (LBNL) on dry ice. Samples for stable isotope analysis were filtered into evacuated 125 mL vials (no headspace) as soon as possible after sample collection. Samples for isotope analysis or hydrocarbon profiling were kept at 4°C and shipped to LBNL. The Prince Creek Formation water (PC), used for make-up water for secondary recovery, and the following oil reservoirs were sampled in 2011: Schrader Bluff (SB1 and SB2), Kuparuk (K1,) Sag River (SR1). None of these production wells were considered soured. See Figure 1 for a schematic of relative formation position and sample IDs.

In 2013, paired wells were selected from Kuparuk and Ivishak reservoirs specifically to examine differences between wells considered not soured (K2, I1) or soured (K3, I2). Samples were collected in 5-gallon containers and shipped to LBNL at ambient temperature, stored at 4°C upon arrival and passed through 0.22 micron filters (Sterivex, Millipore Corp., Billerica, MA,) within 4 days of initial collection. Samples for sulfur and oxygen isotope analysis were processed once sample containers arrived at LBNL. Sample K2 was emulsified, so it was held at 60°C overnight before shipment to LBNL in an attempt to break the emulsion. As it was still emulsified upon arrival at LBNL, salt was added to aliquots of sample to break the emulsion before filtering (see Supplementary Material for additional details). Table S1 contains a summary of analyses performed for each sample, as described below.

### Chemical and isotopic analyses

Hydrocarbon profiles of oil samples from the Schrader Bluff, Kuparuk, and Sag River Formations collected during 2011 were determined by GC-MS at the University of the Pacific. Carbon isotope analyses of oil separates (bulk oil and C8 through C25 hydrocarbons) from the same three formations were performed at the Center for Isotope Geochemistry (CIG) at LBNL using standard analytical techniques. The concentrations of chloride and sulfate, the concentrations and δ^13^C of dissolved inorganic carbon, methane and C2–C5 alkanes, and the δD of methane dissolved in formation water samples from the Prince Creek Formation and the Schrader Bluff, Kuparuk, and Sag River reservoirs were also analyzed at CIG. In addition, the hydrogen and oxygen isotopic ratios of the water in these samples plus additional samples of formation waters from the Kuparuk and Ivishak Formations collected in 2013 were analyzed at the Laboratory for Environmental and Sedimentary Isotope Geochemistry (LESIG) at the University of California Berkeley. The δ^34^S of sulfate in several of the 2013 samples also were analyzed at LESIG. The isotopic compositions are reported using the per mil (δ) notation relative to internationally-accepted standards. More detail about these analytical methods and their precisions are included in the Supplementary Material.

### DNA extractions

Genomic DNA was extracted from filters using a modified Miller Method (Miller et al., 1999). One half of each filter was cut into small pieces and placed in a Lysing Matrix E tube (MP Biomedicals, Solon, OH). 300 μL of Miller phosphate buffer and 300 μL of Miller SDS lysis buffer were added and mixed. 600 μL phenol:chloroform:isoamyl alcohol (25:24:1) was then added, and the tubes were bead-beat at 5.5 m/s for 45 s in a FastPrep instrument (MP Biomedical, Santa Ana, CA). The tubes were centrifuged at 10,000 × g for 5 min. at 4°C. 560 μL of supernatant was transferred to a 2-mL tube and an equal volume of chloroform was added. Tubes were mixed and spun at 10,000 × g for 5 min. 480 μL aqueous phase was transferred to another tube, and two volumes of Solution S3 (MoBio, Carlsbad, CA) was added and mixed by inversion. The rest of the purification procedures followed the instructions in the MoBio UltraSoil DNA extraction kit. Samples were recovered in 60 μL Solution S5 and stored at −20°C.

### PhyloChip analysis

One microliter of DNA template was used in each of eight tubes for bacterial 16S rRNA gene amplification using primers 27F (5′- AGAGTTTGATCCTGGCTCAG -3′) and 1492R (5′- GGTTACCTTGTTACGACTT -3′) in a temperature gradient PCR (48–58°C). Two microliters of template were used for archaeal amplifications using primers 4Fa (5′- TCCGGTTGATCCTGCCRG -3′) and 1492R. For amplifications, 25 μL reactions were prepared as follows (final concentrations): 1x Ex Taq Buffer with 2 mM MgCl_2_, 200 nM each primer (27F and 1492R), 200 μM each dNTP (TaKaRa Bio, Inc., through Fisher Scientific, Pittsburg, PA), 25 μg bovine serum albumin (Roche Applied Science, Indianapolis, IN), and 0.625 U Ex Taq (TaKaRa Bio). DNA was amplified using an iCycler (Bio-Rad, Hercules, CA) and the following thermocycling conditions: 95°C for 3 min. for initial denaturation, 30 cycles of 95°C for 30 s., 48–58°C (annealing temperature gradient, 8 temperatures per sample) for 30 s., and 72°C for 2 min., and then final extension for 10 min. at 72°C. PCR products from the 8-tube annealing temperature gradient for a sample were combined and the total volume was concentrated to 26 μL using a MinElute PCR purification kit (Qiagen, Valencia, CA). One microliter of purified, concentrated PCR product was quantified on a 2% agarose E-gel using the Low Range Quantitative DNA Ladder (Invitrogen, Carlsbad, CA).

Samples for PhyloChip analysis were prepared and processed similarly to Brodie et al. (2006, 2007) and DeSantis et al. (2007). Five hundred nanograms of bacterial PCR product or 100 ng archaeal PCR product was applied to a G3 PhyloChip™ (Second Genome, South SF, CA) following previously described procedures (Hazen et al., 2010). Briefly, the 16S rRNA amplicons and a mix of amplicons at known concentrations (spike-mix) were combined, fragmented using DNAse (Invitrogen, Carlsbad, CA), and biotin-labeled. Labeled products were hybridized overnight at 48°C and 60 rpm. The arrays were washed, stained, and scanned as previously described. Details on probe selection, probe scoring, data acquisition, and preliminary data analysis are presented elsewhere (Hazen et al., 2010). Data obtained from the CEL files (produced from GeneChip Microarray Analysis Suite, version 5.1) were scaled by setting the spike-mix mean intensity to 10,000 to compensate for slight differences in probe responses on different chips. PhyCA was used to process the data with the following parameters: Bacterial array Stage1 cutoffs: pf 0.92, min_q1 0.8, min_q2 0.93, min_q3 0.98; post-Stage 2 cutoffs: min_q1 0.22, min_q2 0.40, min_q3 0.42; Archaeal array Stage 1 cutoffs: pf 0.01, min_q1 0.5, min_q2 0.93, min_q3 0.98; post-Stage 2 cutoffs: min_q1 0, min_q2 0, min_q3 0.1. OTU intensity data were then normalized to total array intensity and ranked separately for each sample for the bacterial and archaeal data sets. For bacteria, OTU affiliated with the following genera were removed before ranking because most were affiliated with human skin and therefore could be contaminants from sampling or processing: *Corynebacterium*, *Staphylococcus*, and *Aquabacterium*. The top 20 ranked bacterial OTUs or the top 10 ranked archaeal OTUs of each sample (highest intensities) were used to evaluate beta diversity across samples. Subsequently, OTUs of bacteria and archaea reported in the literature as being associated with oil reservoirs along with the OTUs included in the top ranked taxa were used to evaluate fold-changes across the sample set, summarized at the level of genus. Functional roles were attributed to genera based on the literature (where available) to further evaluate which taxa may be involved with hydrocarbon degradation, methanogenesis, and sulfide-production. PhyloChip data are available at http://greengenes.lbl.gov/Download/Microarray_Data/Piceno-et-al_oil-reservoirs_2014.zip.

## Results

### Geochemistry and water isotopes

**Figure 2:**
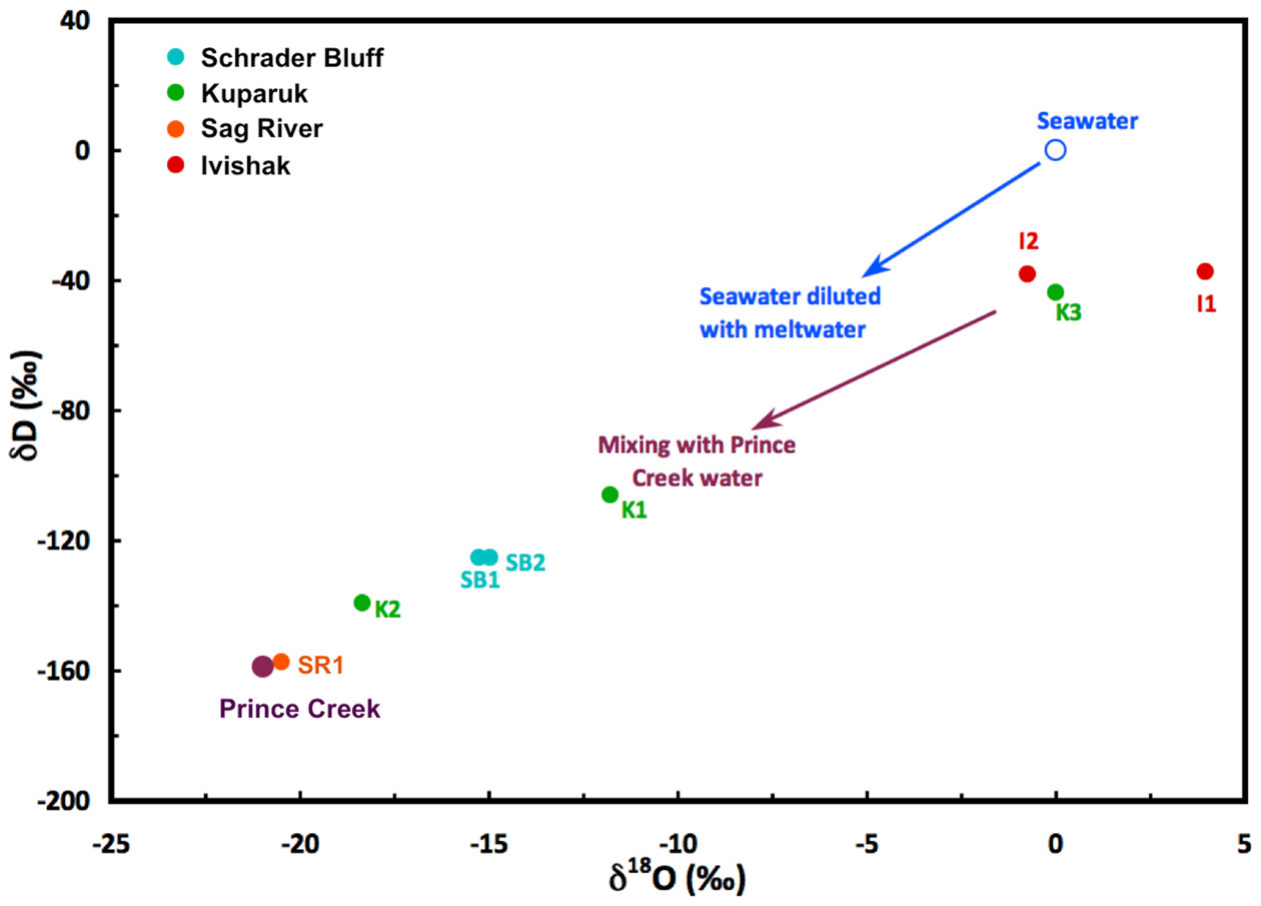
**Hydrogen and oxygen isotope compositions of produced water from North Slope wells**. Waters consist of a mix of reservoir formation waters, Prince Creek Formation water, and/or seawater that were injected to support oil production. North Slope seawater is strongly influenced by melting sea ice and runoff during the summer months.

Concentrations of dissolved chemicals and isotopic compositions showed distinct differences among reservoirs (Table 2). Chloride and sulfate concentrations vary six-fold and >400-fold, respectively, among production wells. The two lowest reservoir chloride concentrations reflect the influence of PC injection water for K2 or lift method for SR1. The complicated injection/production history for the different wells (Table 1) makes it difficult to state unequivocally the reservoir conditions that may be affecting biological activity within the reservoirs. Water isotopes (hydrogen and oxygen) can provide information about the complex mix of fluids that are produced from the wells. Figure 2 is a plot of the measured δD and δ^18^O values for each of the samples. The samples are color coded to reflect the reservoir from which the wells produce and labeled according to the specific well. As noted earlier, Prince Creek water is directly mixed in the production pipe with Sag River produced fluids, so not surprisingly, water isotopes (δD and δ^18^O) from SR1 were nearly identical to that of PC. Prince Creek water also has been injected into the Schrader Bluff and Kuparuk River reservoirs to support production. This influence on these samples can be seen in the isotopic compositions of the water that appear shifted toward the Prince Creek water. The only wells that have had direct seawater injection are I1 and I2. The isotopic composition of seawater on the North Slope is diluted during the summer by a mix of melt water from sea ice, snowmelt, and rainwater. However, the impact of seawater break-through on I2 can be clearly seen by the shift in the δ^18^O of the produced water to a significantly lower value (−1‰ vs. +2 to +4‰), and this well also had the highest sulfate concentration (>600 mg/L). I1 did not show an influence of seawater, which is consistent with a shorter production history and lack of seawater break-through.

**Table 2:** **Chemical and isotopic data for produced water samples**.

**Well Number**	**PC**	**SB1**	**SB2**	**K1**	**K2**	**K3**	**SR1^a^**	**I1**	**I2**
Status^b^	−	NS	NS	NS	NS	S	NS	NS	S
Sulfide^c^ (mg/L)	−	−	−	−	17.5	130	−	14−28	200
DIC (mM)	2.2	47.3	30.1	32.5	−	−	3.3	−	−
DIC δ^13^C (‰)	28.5	15.4	13.8	−2.6	−	−	5.8	−	−
Oil δ^13^C (‰)	−	−29.8	−30.0	−30.3	−30.5	−	−31.3	−	−
CH_4_ (% headspace)	1.4	0.8	1.2	1.3	−	−	1.1	−	−
CH_4_ δ^13^C (‰)	−56.5	−48.0	−49.7	−44.0	−	−	−44.1	−	−
CH_4_ δD (‰)	−259	−267	−281	−178	−	−	−197	−	−
Ethane (ppmv)	90	933	1737	2746	−	−	3562	−	−
Ethane δ^13^C (‰)	−	−25.8	−28.0	−30.3	−	−	−33.8	−	−
Propane (ppmv)	nd	1200	1996	1432	−	−	4622	−	−
Propane δ^13^C (‰)	−	−26.1	−26.7	−29.7	−	−	−33.3	−	−
Butane (ppmv)	nd	453	494	359	−	−	1844	−	−
n−Butane δ^13^C (‰)	−	−27.5	−28.5	−30.7	−	−	−33.1	−	−
Isopentane δ^13^C (‰)	nd	−27.1	−28.5	−31.2	−	−	−32.0	−	−
Water δD (‰)	−159	−125	−125	−106	−139	−44	−157	−38	−38
Water δ^18^O (‰)	−21.0	−15.0	−15.3	−11.8	−18.3	0.0	−20.5	4.0	−0.8
Cl (mg/L)	2444	8170	7050	9366	2092	11,629	2930	11,500	13,118
SO_4_ (mg/L)	1.4	nd	nd	4.2	4.5	11.5	1.8	76	611
δ^34^S (‰)	bd	bd	bd	bd	bd	bd	bd	bd	30.1

−, not available; nd, not detected; bd, below detection.

a Influenced by PC water used for artificial lift.

b NS, non-soured; S, soured as determined by field operators based on historical data.

c Last sulfide measurement available (test separator, gas composition, H_2_S, ASTM D4810-88 (2012–2013).

### Evidence for biodegradation of the oil

**Figure 3:**
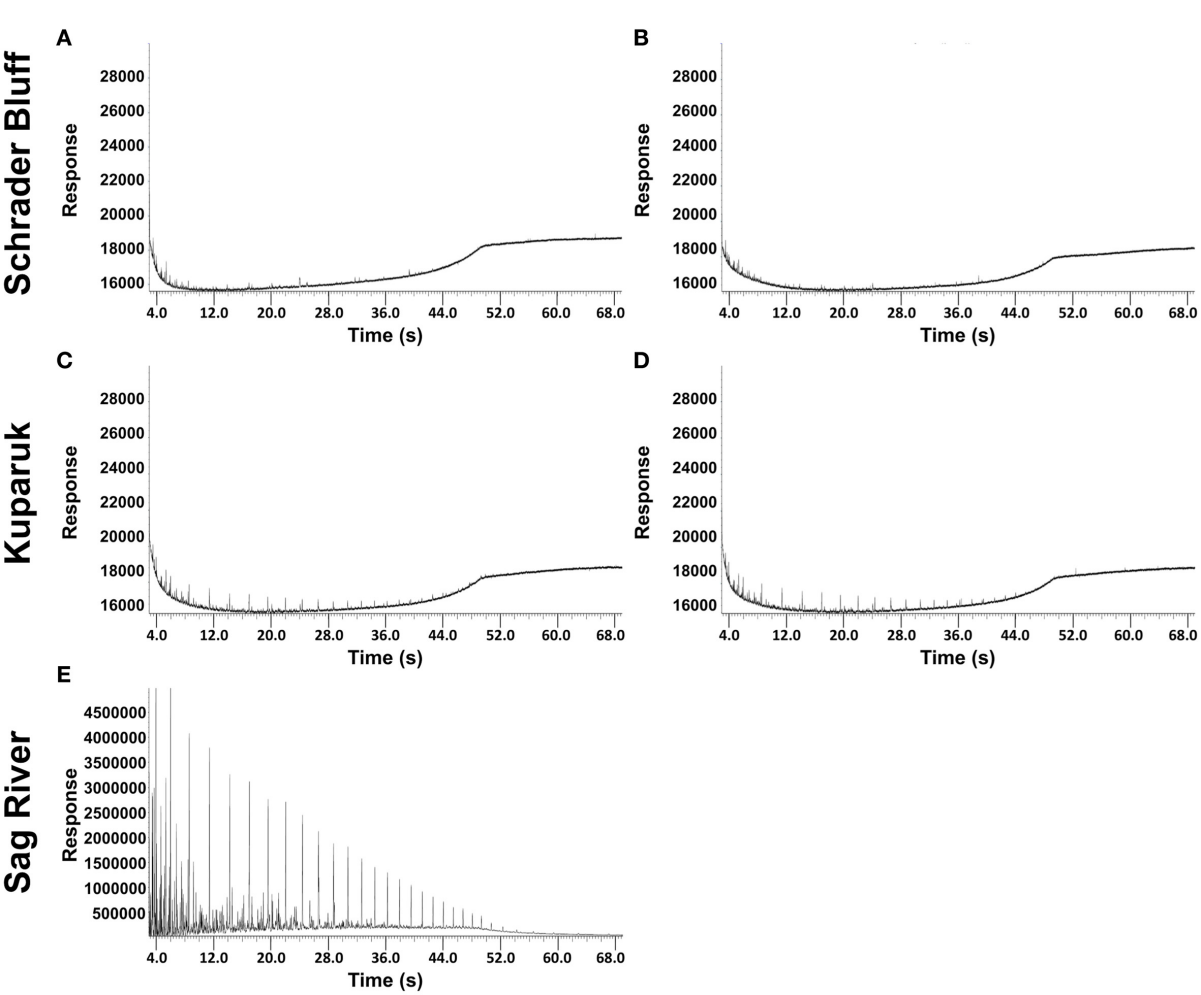
**Chromatograms for volatile fractions of North Slope oil from the Schrader Bluff reservoir (SB1 and SB2 are A,B, respectively), the Kuparuk reservoir (K1 and K2 are C,D, respectively) and the Sag River reservoir (E)**.

GC chromatographs from hydrocarbon analysis show Schrader Bluff oil is strongly depleted in the lighter molecular weight hydrocarbons (<C25) (Figure 3). The Kuparuk sample is somewhat less depleted, and the Sag River sample is rich in low molecular weight hydrocarbons. Microbes metabolizing oil preferentially use lower δ^13^C molecules, resulting in higher δ^13^C values for the residual oil. The δ^13^C values of oil from SR1, K1, K2, SB1, and SB2 were measured, with SR1 (−31.3‰) < K1/K2 (average = −30.4‰) < SB1/SB2 (average = −29.9‰), implying greater degrees of biodegradation with decreasing depth of the reservoirs (Table 2). The δ^13^C values of the lighter hydrocarbons (C8–C25) in the Kuparuk were 2–4‰ higher than in the Sag River samples (the concentrations in Schrader Bluff were too low to analyze), also suggesting significant biodegradation of those components (Figure S2). Additionally, the δ^13^C values of the C2 through C5 alkanes dissolved in the water samples were lowest in the Sag River sample, higher in the Kuparuk sample, and highest in the two Schrader Bluff samples (Figure S3). The shifts to higher δ^13^C values for the alkanes are generally taken as a measure of biodegradation, and have been observed previously for North Slope reservoirs (Masterson et al., 2001).

### Methane and sulfate isotopes

Both product (e.g., methane) and reactant (e.g., sulfate) isotopes can be used to assess microbial activity. Dissolved methane δ^13^C values ranged from −44‰ in the Kuparuk and Sag River samples to −56.5‰ in the PC water sample (Table 2). The δD values of methane showed a similar trend with the highest value in K1 and lowest value in PC. Both carbon and hydrogen isotope data suggest biogenic methane production in the Prince Creek Formation and predominantly thermogenic methane in Kuparuk and Sag River. The δ^34^S value derived from sulfate isotopes changes as sulfate-reducing microorganisms reduce sulfate to sulfide. Sulfate and sulfide concentrations were highest in I2 (Table 2) where seawater break-through has occurred, and only I2 contained enough sulfate to measure the δ^34^S. The δ^34^S of the residual sulfate in the water was +30.1‰, which is higher than the δ^34^S value commonly used for seawater sulfate (+21‰), corroborating significant bio-reduction of sulfate was occurring in the reservoir. The amount of the isotopic shift can be used to estimate the degree of sulfate reduction that occurred. Assuming a fractionation factor of +30‰, a commonly used value for sulfate reduction in natural environments, the measured value of +30.1‰ corresponds to ~25% reduction of the initial sulfate. The measured concentration of sulfate in I2 was 611 mg/L. The water in the sample was estimated to be ~40% seawater based on the water isotope composition, with a sulfate concentration of ~2113 mg/L (taking into account the average of summer (1380 mg/L) and winter (2895 mg/L) measured seawater sulfate concentrations (communication from field operators), due to snow/ice melt). Assuming minimal sulfate in the formation water, the initial sulfate concentration of the water would be ~845 mg/L. 25% reduction would leave a residual sulfate concentration of 634 mg/L, which is very close to the concentration measured in the sample.

### Community structure

**Figure 4:**
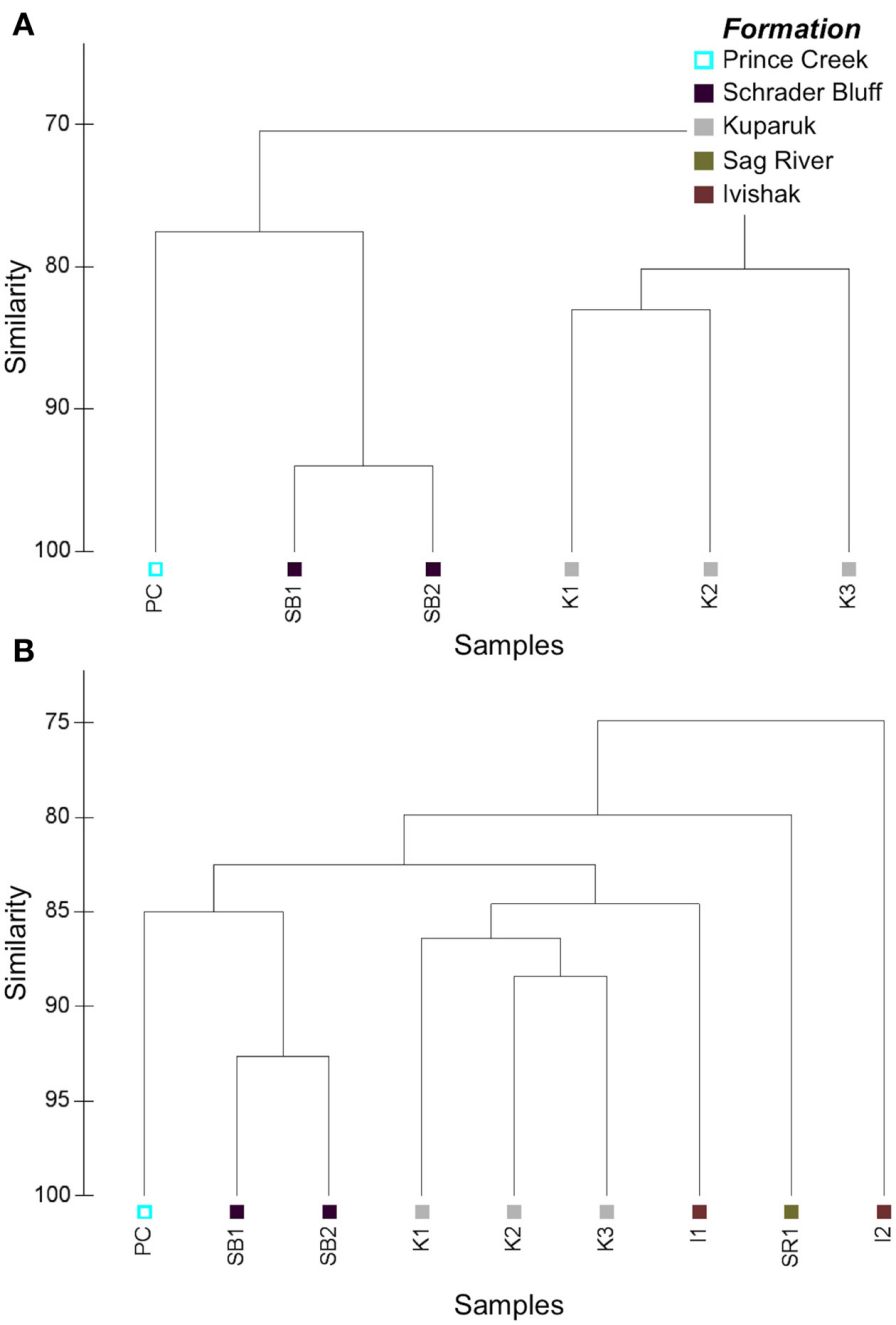
**Cluster plots based on Bray-Curtis similarities of PhyloChip hybridization intensity data used to compare (A) archaeal and (B) bacterial microbial community structures**. Hybridization scores were post-scale-normalized to total array intensity.

PhyloChip analysis of 16S rRNA genes was used to characterize the relative distribution of taxa among samples. Bacterial and archaeal community profiles differed among reservoirs and especially between the soured and non-soured Ivishak wells (Figure 4). Overall microbial community structure was most similar between Schrader Bluff and Prince Creek samples, perhaps due to the cooler formation temperatures. There was greater variability in microbial community structures among the Kuparuk wells, but Kuparuk wells still clustered together. The bacterial communities of the Ivishak wells were very different from one another. Differences in community structures observed among samples were not solely due to wells being located in different fields (Milne Point field vs. Prudhoe Bay fields), as can be observed in Figure 4B where all three Kuparuk samples cluster together (despite the wells being located in two different fields) and one Ivishak sample also clustered with the Kuparuk samples rather than with the other Ivishak sample, despite both Ivishak samples being from the same field. Taxa contributing to the greatest differences across samples are summarized in Tables S2, S3 (including phyla Euryarchaeota, Acidobacteria, Bacteroidetes, Chloroflexi, Cyanobacteria, Firmicutes, Gemmatimonadetes, GN04, Lentisphaerae, OP9, Proteobacteria, Synergistes, Tenericutes, Thermodesulfobacteria, Thermotogae, TM7, and WS3).

OTUs were assigned ranks based on the post-scale-normalized hybridization intensities to account for differences in hybridization intensities and total array intensities. Similar rankings among taxa indicate similar microbial community structures among samples. Several top-ranked bacterial OTUs in the PC sample aligned with *Acetobacterium* (Table S2). Some of the highest ranked bacterial taxa in Schrader Bluff wells (SB1, SB2) were novel members of Phylum OP9 (classified as Class JS1 with both BA021 and SB-45 orders), Phylum GN04, and Phylum Synergistes, and a *Desulfotomaculum* OTU containing a sequence previously recovered from Schrader Bluff by Pham et al. (2009). Several OTUs within the Family Thermoanaerobacteraceae were among the top-ranked bacterial OTUs in Kuparuk samples. K1 also had abundant *Marinobacterium*, and K2 had abundant *Thermodesulfobacteriaceae*. K3 had relatively abundant *Moorella*, *Thermacetogenium*, and unclassified OTUs within Phylum Gemmatimonadetes. SR1 produced water is fairly thoroughly mixed with PC due to the lift method used, explaining the similarity of genomic profiles and water isotope compositions. Because of the direct and unavoidable influence of PC on SR1, incontrovertible results from this sample are limited. In the non-soured Ivishak sample, five of the ten highest ranked taxa were affiliated with *Thermoanaerobacter* and an additional three were aligned to *Halomonas*, whereas *Desulfonauticus* and other SPM were among the highest ranked taxa in the soured I2 sample (Table S2).

Archaea were successfully amplified in Schrader Bluff, Kuparuk, and PC samples, but not from Sag River or Ivishak samples. This does not preclude archaea from being present in Sag River or Ivishak, but we were unable to amplify visible amounts using our PCR primers and amplification conditions. Archaeal communities in Schrader Bluff and PC were distinct from Kuparuk (Figure 4A). The top-ranked archaeal OTU in the Prince Creek Formation sample aligned with *Methanolobus* (a methylotrophic methanogen within the Family Methanosarcinaceae (Penger et al., 2012; Nazaries et al., 2013), an unclassified OTU in Class Thermoplasmata, and *Archaeoglobus* (Table S3). *Methanosaeta* (acetotrophic methanogens), unclassified members of Phylum Thermoplasmata, and several OTUs affiliated with *Methanoculleus* (hydrogenotrophic methanogens) were the top-ranked archaeal OTUs in Schrader Bluff samples. In Kuparuk samples, *Archaeoglobus* and *Thermococcus* were top ranked in K1 and K2, though not K3. *Methanoculleus* was also highly ranked in K1. Seven of the ten highest ranked taxa in K3 were in the Family Methanobacteriaceae (hydrogenotrophic methanogens).

Bacteria and archaea commonly associated with sulfate-reduction were present in all production waters yielding PCR product. Differences in relative abundance of various sulfate-reducers were observed among reservoirs (Table S4), likely related to differences in temperature, salinity, and length of time exposed to high sulfate concentrations. *Desulfosporosinus* had greatest relative abundance in PC, whereas several *Desulfotomaculum* and *Pelotomaculum* OTUs were most abundant in the Schrader Bluff reservoir. Other *Desulfotomaculum* OTUs were more prominent in the hotter reservoirs. Unclassified members in the Family Thermodesulfobacteriaceae had greatest relative abundance in Kuparuk, and *Desulfuromonas*, *Desulfonauticus*, and some OTUs in *Desulfomicrobium* were most prevalent in Ivishak, though only in the soured Well I2. Although sulfate-reducers were present in SR1, all were at low relative abundance. The Kuparuk reservoir samples had the highest relative abundances of OTUs affiliated with the genus *Archaeoglobus*. Also of note, although K3 and I2 had elevated sulfide measurements in produced fluids (Table 2), only I2 had a substantially higher abundance of SPM relative to the paired non-soured well from the same reservoir.

## Discussion

Distinctive microbial communities were present in each of the reservoirs and, in the Ivishak reservoir, prolonged seawater injection had an especially large impact. The taxonomic analysis presented here along with the chemical and stable isotope analyses provide insight into the ecosystem dynamics in multiple reservoirs stratified by depth in this important Alaskan oil reserve. These findings are based on DNA extracted from produced fluids from reservoirs with a complex injection water history or produced with artificial lift and so it is likely some of the over-lapping genomic sequences were present because of the field operating conditions and analytical methods used. A comparative analysis of the four reservoirs is presented with respect to key players in hydrocarbon degradation.

### Fermentation/syntrophy

Fermentative/syntrophic organisms are instrumental in anaerobic crude oil degradation, especially when electron acceptors such as sulfate are in short supply (Callbeck et al., 2013). Kuparuk and Schrader Bluff oil samples showed heavy biodegradation and had numerous fermentative/syntrophic organisms. In particular, *Kosmotoga* and unclassified sequences within Family Thermovirgaceae (within Class Synergistia) had higher relative abundances in Schrader Bluff samples than other samples, and known relatives are capable of fermentation (DiPippo et al., 2009) or synergistic hydrocarbon degradation (Duncan et al., 2009). Additionally, members of the genus *Syntrophus* can degrade fatty acids in syntrophic association with hydrogenotrophic methanogens (Jackson et al., 1999), and OTUs within this genus had high relative abundance in Schrader Bluff samples. Some *Desulfotomaculum* are able to grow syntrophically with hydrogenotrophic methanogens when fermenting propionate (Nilsen et al., 1996b; McInerney et al., 2009; Sieber et al., 2012), and OTUs aligned to *Desulfotomaculum* were among the top ranked OTUs in these samples. Importantly, most the *Desulfotomaculum* and all the *Pelotomaculum* OTUs with greatest relative abundance in the Schrader Bluff samples typically contained sequences recovered from mesophilic environments or enrichments, including an OTU with one clone recovered from the Schrader Bluff reservoir previously (Pham et al., 2009). The obligate anaerobic fermenters *Thermococcus* and *Pyrococcu*s (Jeanthon et al., 2005) were prominent in Kuparuk samples, as were several members of the Thermoanaerobacteraceae (Table S4). The notable differences in the relative abundances of these fermentative populations, despite similar mixed injection water, indicates temperature affects which fermenters are most abundant in the reservoirs.

Samples from the deepest two reservoirs (both roughly 80°C) differed substantially in community structure, as well as injection water history. Sag River has had no water injected, whereas Ivishak has been supported with produced Ivishak water and seawater, the latter being a possible source of new organisms. The Sag River oil sample appeared non-degraded, the isotope data indicated the methane in this sample was thermogenically-produced, and given the temperature is fairly stable at 80°C, there is little to suggest much microbial activity in this reservoir. These findings are consistent with part of the paleosterilization hypothesis (Wilhelms et al., 2001), though the reservoir has not been uplifted beyond 80°C. Though we do not have a hydrocarbon profile for Ivishak, the souring in I2 indicates hydrocarbons are being degraded. It is possible the high temperature (away from the injection wells) and higher salinity of the Ivishak reservoir may exceed physiological tolerances of several fermentative meso- and thermophiles found in the Schrader Bluff and Kuparuk reservoirs, resulting in markedly different microbial communities. *Thermoanaerobacter* OTUs and several OTUs classified within the Halomonadaceae may have been the main fermentative/hydrocarbon degrading bacteria in I1. Isolates related to *Thermoanaerobacter* have been isolated from oil reservoirs previously (Fardeau, 2004; Magot, 2005), and members of *Marinobacter* (Duncan et al., 2009) and *Halomonas* (Mnif et al., 2009) are known to degrade hydrocarbons.

### Methanogenesis

Methanogens require other organisms (e.g., fermenters) to initiate complex organic matter degradation to provide them with one or two carbon compounds, yet fermentation can be thermodynamically unfavorable without syntrophic partners available to consume intermediates, especially hydrogen (McInerney et al., 2009; Sieber et al., 2012; Meslé et al., 2013; Gieg et al., 2014). This syntrophy has been demonstrated through numerous methanogenic hydrocarbon degradation enrichments or co-cultures (Rabus, 2005; Head et al., 2010; Gray et al., 2011; Siddique et al., 2011; Aitken et al., 2013; Gieg et al., 2014). As there was no reported souring for the highly biodegraded Schrader Bluff reservoir samples, methanogenesis is likely the most important terminal electron accepting process in this reservoir (though we cannot rule out the possibility of sulfur cycling within the reservoir). The relative abundances of known acetotrophic methanogens (*Methanosaeta*) were highest in Schrader Bluff compared to other reservoirs (Table S4). Pham et al. (2009) reported acetotrophic methanogens were numerically dominant relative to other methanogens in a fosmid library constructed from produced water samples from the same reservoir, further suggesting acetotrophic methanogenesis is or has been an important methanogenic pathway supported by anaerobic hydrocarbon degradation in Schrader Bluff. Several hydrogenotrophic methanogens also were detected in Schrader Bluff samples. Syntrophy coupled primarily to methanogenesis may be (or have been) a prominent pathway for biogenic methane formation from hydrocarbons in Schrader Bluff.

**Figure 5:**
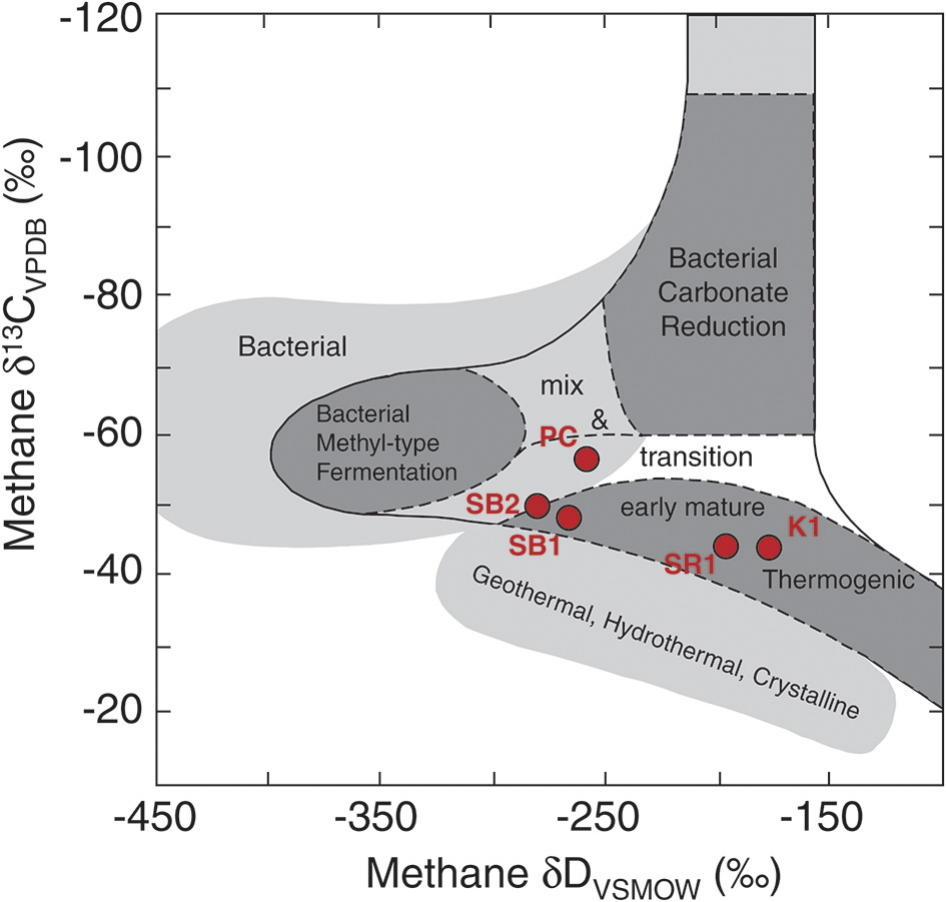
**Carbon and hydrogen isotopic compositions of dissolved methane samples from Prince Creek Formation, and Schrader Bluff, Kuparuk, and Sag River reservoirs**. A shift toward the left for Schrader Bluff samples indicates a significant portion of biogenic methane was produced from the acetoclastic (“fermentation”) pathway. This figure was adapted from Whiticar (1999).

The gas and isotope data provide additional information regarding sources of methane in the reservoirs. The dissolved gas in the Schrader Bluff samples falls in the thermogenic range (Figure S4), but the ratio of methane to (C2+C3+C4) is almost three times higher than it is in the Sag River sample, which is indicative of higher proportions of biogenic methane (Head et al., 2003; Huang and Larter, 2005; Jones et al., 2008). The C1–C4 data (Table 2) also indicate hydrocarbon degradation in the reservoir, and are consistent with metabolites measured by Gieg et al. (2010) in samples collected from Alaskan oil reservoir production wells. The combined δD and δ^13^C values of methane can be used to distinguish different methanogenic pathways. The hydrogen and carbon isotope data for the 2011 water samples were plotted on a figure modified from Whiticar (1999) (Figure 5). The methane in PC is positioned in the “mixed” region of the plot between thermogenic, CO_2_ reduction, and fermentation (acetoclastic) methanogenesis regions. However, these fields are based on the assumption that the source of carbon is CO_2_ or organic matter with a δ^13^C value between −10 and −30‰. The DIC in the Prince Creek water is quite high (+28.5‰) and would yield methane with a higher δ^13^C value. Similarly, the δD value of PC water is −159‰, much lower than the δD value of water in marine environments (~0‰) from which Whiticar established these fields. Based on this, we suggest that methane in the Prince Creek reservoir is primarily produced by CO_2_ reduction. The δ^13^C values of methane are less negative in the deeper reservoirs and more negative in the shallower, more biodegraded reservoirs (Figure S3). Methane from thermogenic sources generally has less negative δ^13^C values than biogenic methane (Whiticar, 1999, and references therein). The δ^13^C values of methane in SB1 and SB2 are ~5‰ lower and the δD values ~75‰ lower than the isotopic compositions of the Sag River sample. If we estimate that about 50% of the methane in the Schrader Bluff samples is biogenic, that gives an approximate isotopic composition for the biogenic component of about −54‰ for carbon and −350‰ for hydrogen, which is well within the field for the isotopic composition of acetoclastic methane (“Fermentation” field of Figure 5).

In contrast to Schrader Bluff, the deeper Kuparuk reservoir sample from 2011 had ratios of C1–C4 compounds indicative of substantially more thermogenic methane. The δ^13^C and δD isotopic signatures of the Kuparuk samples also indicate mostly thermogenic methane. The PCR-amplified genetic signature, however, contained numerous methanogens. As with Schrader Bluff, syntrophy appeared important in the Kuparuk reservoir, though via a different pathway. Methanogens with higher relative abundance in Kuparuk samples were affiliated with *Methanobacterium*, *Methanobrevibacter*, and for K1, *Methanoculleus* (Tables S3, S4), which are hydrogenotrophic methanogens. Members of the genus *Methanoculleus* are involved with methanogenic CO_2_-reduction in syntrophy with bacteria such as *Syntrophus* and *Marinobacter*, both of which were present in this sample. These bacteria also are known to ferment alkanes to acetate (Head et al., 2010), and while Mueller and Nielsen (1996) found acetate was present (8.3–14.7 mM) in produced water sampled from the Kuparuk reservoir, acetate was not used by methanogens in enrichment cultures set up using the same water, suggesting they were hydrogenotrophic methanogens. Complementing this, some OTUs affiliated with *Thermacetogenium*, thermophilic, syntrophic acetate-oxidizers (Duncan et al., 2009) previously reported to have association with hydrogenotrophic methanogens (Sieber et al., 2012), were most abundant in Kuparuk samples. Jones et al. (2008) proposed syntrophic acetate-oxidation could explain their observations in oil-degrading microcosms whereby the acetate from alkane degradation was oxidized to CO_2_ and hydrogen, supporting the numerically dominant hydrogenotrophic methanogens and producing isotope data consistent with methane generated substantially through CO_2_ reduction. Together, this further supports the idea that hydrogenotrophic methanogens play a more prominent role in Kuparuk hydrocarbon transformation processes than do acetotrophic methanogens, perhaps reflecting temperature optima or limitations of different methanogen populations (Meslé et al., 2013).

We were unable to amplify archaea from Ivishak and Sag River samples. Stetter et al. (1993) cultured hyperthermophilic archaea from Alaskan oil reservoirs, but they were not methanogens. Stetter et al. (1986) note two extreme thermophilic methanogens (*Methanothermus* and *Methanococcus*) have been isolated from other environments, and Orphan et al. (2000) were able to culture methanogens at 80°C from oil production water samples, but our amplification reactions yielded no visible PCR product on gels.

### Sulfidogenesis

Hydrocarbon degradation by SPM can happen by several routes (Stetter and Huber, 1999; Gieg et al., 2011; Callbeck et al., 2013) and could lead to reservoir souring. Two production wells were sampled because they had sulfide levels indicative of souring. In the soured Kuparuk well (K3), potential sulfidogen OTUs with greater relative abundance than in K2 include an unclassified Anaerobaculaceae, an unclassified Synergistaceae, and OTUs classified within the genus *Thermoanaerobacter*. *Anaerobaculum* (within the Anaerobaculaceae) can produce sulfide from thiosulfate, sulfur, and cysteine (Duncan et al., 2009), and isolates of *Thermoanaerobacter* can respire thiosulfate, so these or other compounds may contribute to souring, as was suggested by Orphan et al. (2000) for other oil reservoirs. It is also possible the injection water for K3, which includes water recycled from the Ivishak Formation, contains sulfate and so sulfate-reduction may be increased relative to other Kuparuk wells. There were, however, other sulfidogens (e.g., *Archaeoglobus* and unclassified Thermodesulfobacteriaceae) with lower relative abundances in K3 than K2. While this was unexpected, it is not unique; Nilsen et al. (1996a) observed something similar in an oil field sample containing too few SPM to be detected by fluorescent antibodies yet having the highest concentration of hydrogen sulfide in their sample set. Future activity-based assays, such as would be available from mRNA (given sufficient sampling volumes) could help decipher the actual processes *in situ*.

Seawater has been injected for more than 10 years in the Ivishak Formation, and I2 has several SPM OTUs with higher relative abundances compared to I1. The OTU with greatest relative abundance in I2 (12-fold higher than other samples) was affiliated with *Desulfonauticus autotrophicus*, a thermo- and halophilic bacterium originally isolated from an oil production separator in Hamburg, Germany (Mayilraj et al., 2009). *D. autotrophicus* uses only hydrogen and formate as electron donors and a variety of sulfur compounds, including sulfate, as electron acceptors. A *Desulfonauticus* sp. sequence also was recovered recently from a hyperthermophilic Australian oil reservoir (Li et al., 2013), indicating it may be a globally-distributed oil reservoir inhabitant. Interestingly, an unclassified Syntrophorhabdaceae was also identified in I2 and may have served as the hydrogen source in a syntrophic interaction. The other two most enriched sulfide-producers were affiliated with *Desulfomicrobium* and *Desulfuromonas* (which reduces sulfur to sulfide). Other potential sulfate-reducers were affiliated with *Desulfocapsa*, *Desulfovibrio*, and an unclassified member of the Desulfobacteraceae. We did not detect *Archaeoglobus*, but altering the PCR conditions may allow for their detection if present. Sample I1 did not have sulfide values indicative of souring, but sulfate was measured in the produced water and small amounts of sulfide had been measured recently. Sulfate reduction (or other means of sulfide production) within the reservoir may be occurring with potential re-oxidation or immobilization of most of the sulfide. One OTU with higher relative abundance in I1 compared to I2 is classified within the Sulfobacillaceae (YNPFFP6) and so may be capable of sulfide-oxidation. Monitoring sulfate concentrations of both injected and produced water may help determine the extent of possible sulfur cycling within the reservoir and help predict the timing of the expected souring.

Several SPM also were detected in both Schrader Bluff samples. As neither sample is considered soured, the diversity of SPM was unexpected. It is possible the DNA for these organisms was carried over from other reservoirs because the injection water is a mixed water source and the extracted DNA was PCR-amplified. It is also possible these microbes are living by means other than sulfate-reduction. For example, Muyzer and Stams (2008) state, “Although named after their ability to use sulfate as a terminal electron acceptor, sulfate reducers can use many other electron acceptors for growth and can ferment substrates in the absence of inorganic electron acceptors. Therefore, the occurrence of high numbers of SRB in an environment does not necessarily reflect the occurrence of sulfate reduction in that environment, ….” One example from Schrader Bluff is *Desulfotomaculum*, commonly associated with sulfate-reduction, yet some members [e.g., especially those in subcluster Ih (a subgroup based on 16S rRNA gene sequence)] have been shown to live syntrophically and appear to lack the ability to respire sulfate (Imachi et al., 2006; Plugge et al., 2011). As noted earlier, the trends for *Desulfotomaculum* OTUs that were most abundant in Schrader Bluff samples were different from other *Desulfotomaculum* OTUs containing sequences recovered from thermophilic environments and having greatest relative abundances in the deeper (hotter) reservoirs (Table S4). Both the current study and Pham et al. (2009) found several other sulfidogens in Schrader Bluff also, though Pham et al. did not detect *Archaeoglobus*, perhaps because the two studies used different amplification conditions. While identifying potential sulfidogens in produced water samples using ribosomal gene sequences provides information about some metabolisms potentially occurring in the reservoirs, better information about activity will have to await RNA or protein-based analyses in the future.

## Concluding remarks

The four Alaskan North Slope oil reservoirs differed in microbial community structure and geochemical isotopic signatures in relation to reservoir temperature and injection water sources. Reservoir conditions, especially temperature, influenced microbial community structures of the produced fluids, and the effect of long-term seawater injection was even greater. Fermentative bacteria like *Kosmotoga* were more prevalent in the cooler Schrader Bluff samples, whereas *Thermotoga*, the Thermococcaceae, and several members of the Thermoanaerobacteraceae had greatest relative abundance in the warmer Kuparuk samples. Members of the genus *Thermoanaerobacter* also showed high relative abundance in the non-soured, hyperthermic Ivishak sample. Acetotrophic methanogens were most prominent in Schrader Bluff, hydrogenotrophic methanogens were most abundant in the Kuparuk reservoir, and no methanogens were detected in the deepest (hottest) two reservoirs. Potential sulfide-producing organisms showed similar reservoir-dependent differences in relative abundance, but distributions of SPM were not linked with souring. Sulfate availability due to extensive seawater injection and recycling of that produced water appeared to be an important determinant of souring in Ivishak and Kuparuk reservoirs. Ribosomal RNA gene analysis permits a first-level assessment of genetic potential based on what is already known about organisms. A more detailed analysis of genetic potential can be derived from metagenome analysis, such as has been performed recently for other hydrocarbon-containing systems or isolates (Abbai and Pillay, 2013; An et al., 2013; Dodsworth et al., 2013; Tan et al., 2013; Embree et al., 2014; Lewin et al., 2014; Sierra-García et al., 2014). Ongoing metagenome analysis of several samples from the current study, coupled with isotope geochemistry, is expected to improve our understanding of what microbes, especially novel microbes, may do in these reservoirs and enhance the predictiveness of reservoir souring.

### Conflict of interest statement

Craig Graff and Jiabin Han are employees at BP. Nothing presented in the manuscript poses a conflict of interest. All other authors declare that the research was conducted in the absence of any commercial or financial relationships that could be construed as a potential conflict of interest.
