# Supplementary material for: Temperature and injection water source influence microbial community structure in four Alaskan North Slope hydrocarbon reservoirs
Source: Front Microbiol. 2014 Aug 7;5:409. doi: 10.3389/fmicb.2014.00409 (PMC4124708; doi:10.3389/fmicb.2014.00409)
Supplement: Supplementary file 1 [file Presentation1.PDF]

Supplementary Material

Temperature and injection water source influence microbial community structure in four Alaskan North Slope hydrocarbon reservoirs.

Authors: Yvette M. Piceno[1,2], Francine C. Reid[1,2], Lauren M. Tom[1,2], Mark E. Conrad[1,2], Markus Bill[1,2], Christopher G. Hubbard[1,2], Bruce W. Fouke[2,3], Craig J. Graff[4], Jiabin Han[4], William T. Stringfellow[1,2,5], Jeremy S. Hanlon[1,5], Ping Hu[1,2], Terry C. Hazen[6], and Gary L. Andersen[1,2*]

[1] Earth Sciences Division, Lawrence Berkeley National Laboratory, Berkeley, CA, USA
[2] Energy Biosciences Institute, Berkeley, CA, USA
[3] Department of Geology, University of Illinois at Urbana-Champaign, Urbana-Champaign, IL, USA
[4] Production Chemistry, BP Exploration, Anchorage, AK, USA
[5] Ecological Engineering Research Program, University of the Pacific, Stockton, CA, USA
[6] Department of Civil & Environmental Engineering, University of Tennessee, Knoxville, TN, USA

[*]Correspondence:
Dr. Gary L. Andersen
Center for Environmental Biotechnology
Ecology Department
Lawrence Berkeley National Laboratory
1 Cyclotron Rd., MS 70A-3317H
Berkeley, CA, 94720, USA
GLAndersen@lbl.gov
Office: (510) 495-2795

Materials and Methods

Field filtering method

Water was siphoned out of 5-gallon oil collection drums using plastic tubing and a 60mL syringe. Tubing was kept submerged so as to not disturb the oil/water interphase, and syringe was used to collect 60mL of water at a time. Maximum volume of water was collected from each sample into 1L bottles. After chemical analyses, all remaining water was filtered.
A Gast® Vacuum Pressure Pump (25.5” Hg) was used to filter water through Pall MicroFunnel 0.22µm cup filters attached to a three-place filter manifold. Water was filtered through a given filter until it was gone or until the filter clogged. When a filter clogged, filtering was continued with another filter. All filters were stored and then shipped to LBL on dry ice.

Breaking the emulsion in the K2 produced fluid

Sample K2 was emulsified and so could not be filtered at the field lab in 2011 when first collected. In 2013, the well was resampled and the sample shipped to LBNL where both salt addition and centrifugation were used to separate the water phase from the oil. 5M NaCl was

added to aliquots (< 1500 mL each) of sample K2 to bring salinity up to 2%. This caused the phases to break, but phases were not completely separate. The sample was then centrifuged in ~300mL aliquots at 500 x *g* for 10 minutes to further separate the phases. After centrifugation, water was siphoned and filtered in the same way as the other 2013 samples.

Chemical and isotopic analytical techniques
To determine the low molecular weight (<C25) hydrocarbon profile of oil samples from three formations (Schrader Bluff, Kuparuk, Sag River), 100 ppm of oil was dissolved in methylene chloride. One µL injections were made with an automated liquid sampler into an Agilent 6890N gas chromatograph with an FID detector (30 m * 250 µm Supelco SLB-5ms capillary column (Supelco 28471-u) using a method adapted from the UNEP protocol (UNEP/IOC/IA, 1992). The inlet was set to splitless and it's temperature was held at 250°C. The carrier gas was helium and had a constant flow of 1.8ml/min. The oven was programmed to start 50°C and hold for one minute before increasing at 5°C/min to 290°C and holding for 20 minutes, giving a total run time of 69 minutes. The major inorganic anion concentrations in both the 2011 and 2013 water samples were measured using a Dionex IC 1500 (only chloride and sulfate were detectable).

$\delta^{13}C$ of the bulk oil samples were analyzed using an ECS 4010 Elemental Analyzer (Costech Analytical Technologies Inc., Valencia, USA) coupled to a Thermo Scientific Delta V™ Plus isotope ratio mass spectrometer (Delta V) at the Center for Isotope Geochemistry (CIG), Lawrence Berkeley National Laboratory. Approximately 1 µL of oil was placed in a 4x6 mm Costech tin capsule and loaded into a zero blank auto-sampler. The reproducibility of these analyses is ±0.1‰ (1σ). Concentrations and $\delta^{13}C$ of CH_4 were analyzed using a Tracegas™ pre-concentrator system to convert CH_4 to CO_2 before introducing it to a Micromass JA Series Isoprime isotope ratio mass spectrometer (Isoprime). The concentrations of CH_4 were determined by comparing the peak area of the sample to a daily standard calibration curve. δD of CH_4 in the samples were analyzed by flushing headspace gas through the sample loop of a 6-port valve connected to a HP-Molesieve capillary column (30 m x 0.32 mm x 12.0 µm) of a Thermo Scientific Trace GC Ultra (Trace GC) interfaced with the Delta V. The precision of the measured δD and $\delta^{13}C$ values of the CH_4 is ± 5‰ and ± 0.5‰ (1σ), respectively. The precision on CH_4 concentrations is ± 5% of the measured values. Concentrations and $\delta^{13}C$ of C2-C5 alkanes were measured by injecting from 120 µL to 500 µL of the headspace gas sample into a He stream injector connected to a liquid N_2 stainless loop trap mounted on a 6-port valve. During the sample transfer, water and CO_2 were removed using magnesium perchlorate and Ascarite II (sodium hydroxide coated non-fibrous silicate). The pre-concentrated samples were injected into a gas chromatograph (Hewlett Packard 6890 GC) by switching the 6-port valve and heating the loop with a heat gun. C2 –C5 alkanes were separated chromatographically in a GasPro fused silica capillary column (60 m x 0.32 mm) and combusted to CO_2 at 850 °C in a capillary quartz tube loaded with Cu, and Pt wires. The carbon isotope ratios of the resulting CO_2 were analyzed using the Isoprime. The concentrations were determined using the mass 44 (CO_2) peak area of the combusted alkanes. The precision for the concentrations is ±10% of the measured values and for the $\delta^{13}C$ values is ±1‰ (1σ). $\delta^{13}C$ analyses of C8-C25 alkanes were analyzed by injecting 0.5 µL of sample at 1:9 analyte to CH_2Cl_2 ratio into a Trace GC. C8-C25 were separated

chromatographically on an HP-5 fused silica capillary column (30 m x 0.25 mm) and combusted to CO_2 at 1000 °C in a capillary ceramic tube loaded with Ni, Cu, and Pt wires, water was removed, and the carbon isotope ratios were measured with the DeltaV. The reproducibility of the $\delta^{13}C$ values of the individual compounds was ±0.5‰ (1σ). Total dissolved inorganic carbon of water was measured in 6 to 60 μL aliquots extracted with an airtight syringe and injected into Helium flushed 5.9 ml Labco exetainer® vials containing 0.2 mL of 99.5% phosphoric acid (H_3PO_4). The CO_2 resulting from the reaction of DIC with H_3PO_4 was analyzed using a headspace autosampler (Gilson, Villiers-le-Bel, France) linked to a Tracegas™ preconcentrator interfaced to the Isoprime. Sample DIC concentrations were determined using the mass 44 (CO_2) peak area. With this technique, the precision of the concentration is ±0.5 mM (1σ) and the $\delta^{13}C$ value is ±0.25‰ (1σ).

Oxygen and hydrogen isotope of water samples were analyzed at the Laboratory for Environmental and Sedimentary Isotope Geochemistry (LESIG) at the University of California Berkeley using established methods. δD analyses were done with the Eurovector model 3028 elemental analyzer fitted with a Cr-reduction furnace interfaced to a GV Isoprime isotope ratio mass spectrometer. $\delta^{18}O$ analyses of the water were made by equilibrating the oxygen in CO_2 with a sample of the water in a multi-prep system interfaced with the Isoprime. The precisions of the δD and $\delta^{18}O$ analyses are ±0.3‰ and 0.05‰ (1σ), respectively. Sulfur isotope analyses of sulfate were also analyzed at LESIG. The sulfate in the water was precipitated as $BaSO_4$ by addition of a 1 N solution of BaCl to the water samples. The $BaSO_4$ precipitates were filtered, dried and analyzed using established methods. V_2O_5 was added to the samples for sulfur isotope analyses to aid with combustion. Precision is ±0.2‰ (1σ) for sulfur isotope analyses.

All isotopic data are reported using the conventional delta notation where:

$$\delta^{13}C_{VPDB}\ (‰) = [(^{13}C/^{12}C)_{sample}/(^{13}C/^{12}C)_{standard} - 1] \times 1000$$

Data are reported relative to Vienna PeeDee Belemnite (VPDB) for carbon, Vienna Standard Mean Ocean Water (VSMOW) for hydrogen and water, and Vienna Canyon Diablo Troilite (VCDT) for sulfur.

Supplementary Tables and Figures

Table S1. Sample information and analyses performed (X) on produced water.

Formation/ Reservoir	Well	Status[2]	Year Sampled	HC analysis	Isotope analysis[3]	Vol (mL) extracted for gDNA	gDNA recovered (ng/uL)	Array analysis[4]
Prince Creek	PC[1]	-	2011	-	X	425	0.08	B, A
Schrader Bluff	SB1	NS	2011	X	X	125	2.18	B, A
Schrader Bluff	SB2	NS	2011	X	X	80	5.69	B, A
Kuparuk	K1	NS	2011	X	X	100	< 0.01	B, A
Kuparuk	K2	NS	2013	X	X	3125	0.76	B, A
Kuparuk	K3	S	2013		X	4190	2.23	B, A
Sag River	SR1	NS	2011	X	X	350	< 0.01	B
Ivishak	I1	NS	2013		X	5000	0.11	B
Ivishak	I2	S	2013		X	2935	< 0.01	B

[1] PC, Injection water supporting secondary recovery in Schrader Bluff and Kuparuk reservoirs and used for artificial lift for SR1.
[2] NS, non-soured; S, soured as established by field operators using historical data.
[3] Not all analyses were possible for both sampling years. See Table 2 for detailed data obtained.
[4] Bacterial (B) PCR product or archaeal (A) PCR product assayed separately by PhyloChip microarray analysis. Not all samples yielded archaeal PCR product.

Table S2. Bacterial taxa comprising the top 20 OTU in each sample from PhyloChip post-scale-normalized hybridization intensity scores. Ranks 1-10 in each sample are bolded and highlighted. [See pdf appended at end of document.]

Table S3. Archaeal taxa comprising the top 10 OTU in each sample from PhyloChip post-scale-normalized hybridization intensity scores. Ranks 1-10 in each sample are bolded and highlighted. [See pdf appended at end of document.]

Table S4. PhyloChip data summarized for bacteria and archaea commonly found in oil reservoirs. Taxa with the highest ranked OTU hydribization scores also were included (top 20 OTU for bacteria or top 10 OTU for archaea according to relative probe intensity). Data bars represent post-scale-normalized OTU intensity values and are scaled within the group across samples. Taxa within a domain are grouped by the primary functional role commonly associated with a lineage as reported in the petroleum reservoir literature or broader hydrocarbon or marine literature as indicated. Many organisms are metabolically flexible and so potentially participate in multiple pathways, depending on local conditions. Common alternative roles are indicated when known.
[See pdf appended at end of document.]

Figures

Figure S1 – Map of the fields (Milne Point and Prudhoe Bay units) and generalized stratigraphy of formations sampled (Ivishak, Sag River, Kuparuk, Schrader Bluff, and Prince Creek) on the Alaskan North Slope.

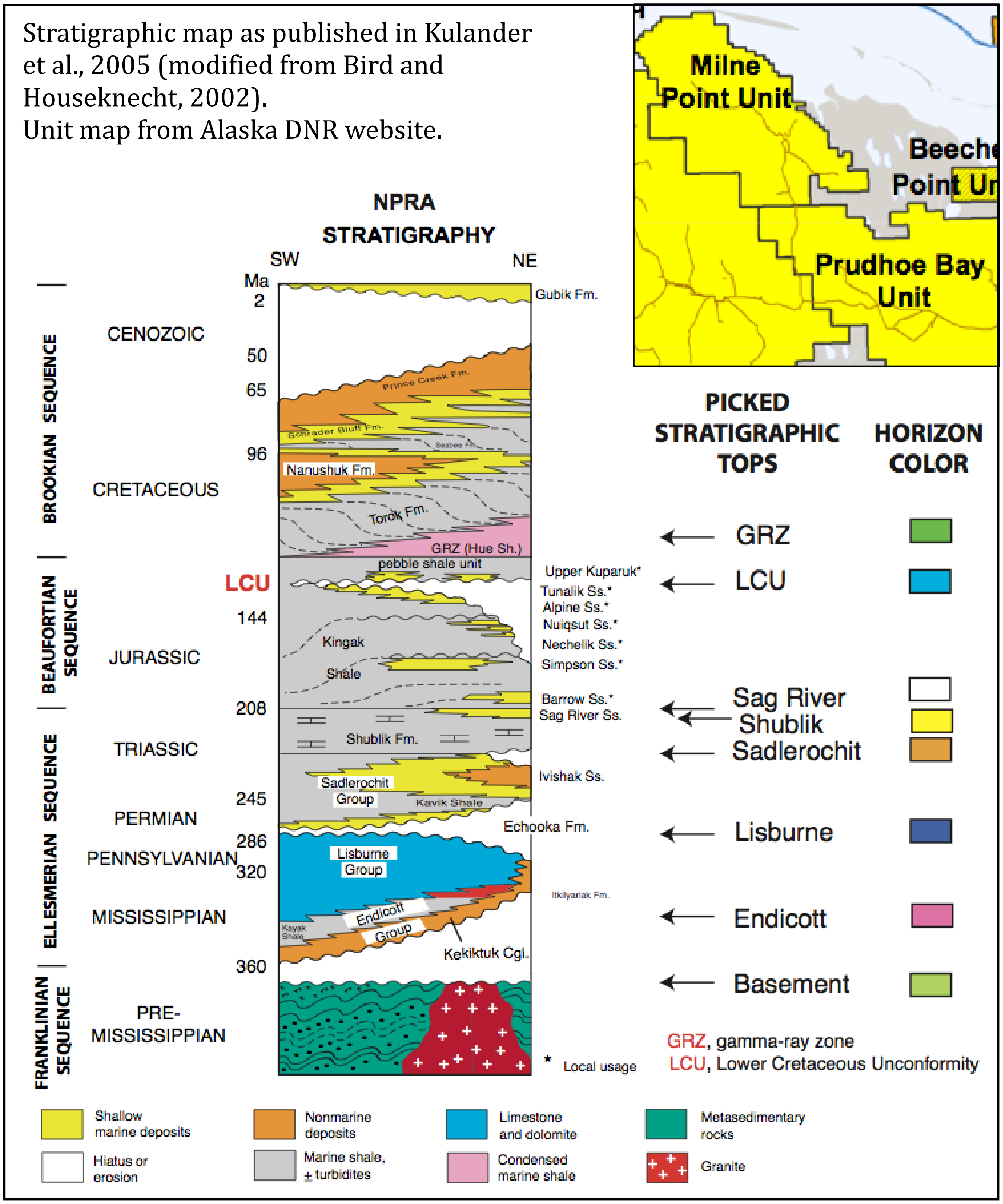

Figure S2 - Compound specific carbon isotope data for lighter hydrocarbons in the Kuparuk and Sag River reservoirs.

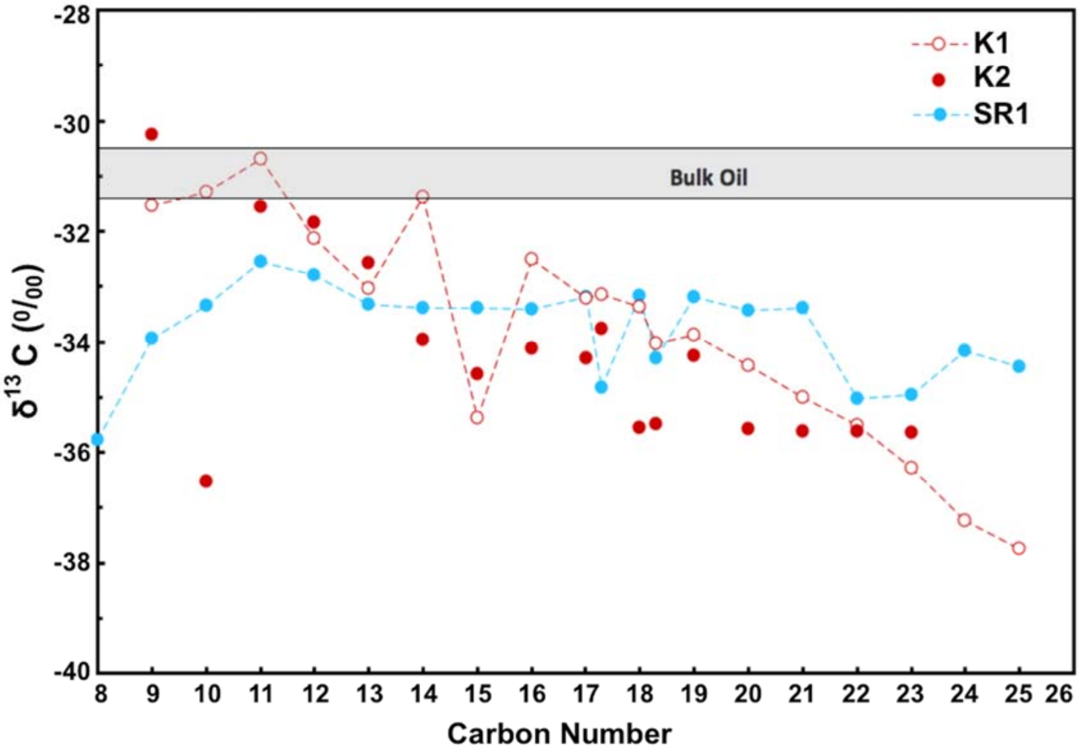

Figure S3 - Carbon isotope compositions of C1 through C5 alkanes dissolved in production water samples from the Schrader Bluff, Kuparuk and Sag River reservoirs. Higher values for C2 through C5 in the Kuparuk and Schrader Bluff signify an increasing level of microbial metabolism of those samples. Lower methane $\delta^{13}C$ values in the Schrader Bluff samples indicate a component of biogenic methane in the samples.

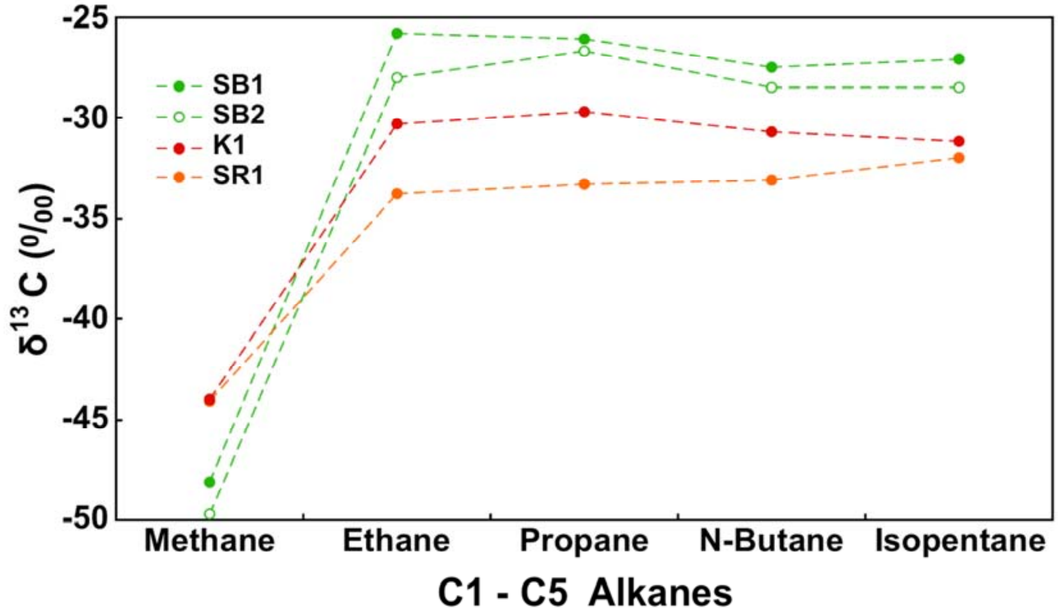

Figure S4 - Carbon isotope compositions of methane plotted versus the ratio of concentrations of methane to C2 through C4 compounds dissolved in water samples from Prince Creek, Schrader Bluff, Kuparuk, and Sag River reservoirs.

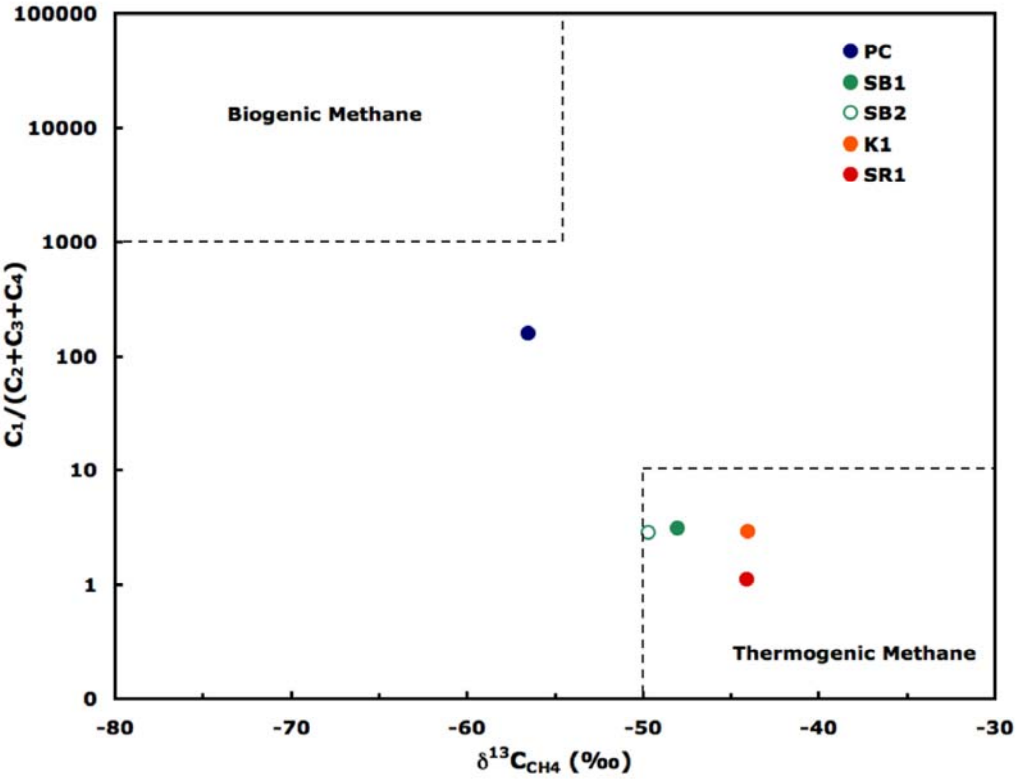

Table S2. Bacterial taxa comprising the top 20 OTU in each sample from PhyloChip post-scale-normalized hybridization intensity scores. Ranks 1-10 in each sample are bolded and highlighted.

Phylum	Class	Order	Family	Genus	OTU_ID	PC	SB1	SB2	K1	K2	K3	SR1	I1	I2
Acidobacteria	Acidobacteria	Acidobacteriales	Acidobacteriaceae	unclassified	50993	190	356	361	53	151	178	228	298	**7**
			Propionibacteriaceae	Propionibacterium	40264	85	18	19	29	40	28	159	39	27
					41012	49	15	15	64	121	64	125	75	142
			Streptosporangiaceae	Nonomuraea	41276	424	426	121	15	178	79	471	526	53
Bacteroidetes	Bacteroidia	Bacteroidales	Porphyromonadaceae	Dysgonomonas	68707	885	228	339	108	257	1168	19	1218	1186
			RikenellaceaeII	unclassified	69770	14	13	11	**7**	78	39	**10**	56	**2**
Chloroflexi	Anaerolineae	Anaerolineales	Anaerolinaceae	A4b	53690	54	42	64	127	27	31	14	35	48
				H39	58135	12	94	72	68	81	204	256	393	25
				T78	58039	**2**	16	32	1190	1134	971	411	1096	845
					58522	19	66	43	234	214	248	132	241	350
	Chloroflexi-4	unclassified	unclassified	sfB	52123	75	22	65	114	78	45	119	294	**4**
Cyanobacteria	Chloroplast	Euglenozoa	unclassified	sfA	75099	617	17	24	250	394	114	314	682	847
Cyanobacteria	Oscillatoriophycideae	Chroococcales	Microcystaceae	Microcystis	53792	242	360	376	200	44	15	358	213	29
Firmicutes	Bacilli	Bacillales	Bacillaceae	Bacillus	49332	353	137	54	**5**	107	87	704	89	191
			Pasteuriaceae	unclassified	49517	130	109	52	51	40	40	322	98	18
			Planococcaceae	Planococcus	42701	282	92	37	12	70	61	572	85	579
		Lactobacillales	Lactobacillaceae	Lactobacillus	72809	99	147	209	263	153	111	16	328	137
			Streptococcaceae	Streptococcus	44217	**8**	23	**10**	14	23	20	**5**	55	261
					44835	**6**	319	360	280	112	126	175	704	651
	Clostridia	Clostridiales	Eubacteriaceae	Acetobacterium	69405	**4**	165	162	435	247	190	**3**	263	252
					69638	137	823	1035	1187	952	1099	15	1181	1122
					69808	**10**	503	793	1167	1191	1094	**8**	1189	899
					69813	**5**	20	12	79	167	98	**1**	70	185
					69835	**1**	86	244	603	518	628	**2**	952	709
					69902	51	380	494	732	416	559	**9**	745	538
				unclassified	69880	**10**	74	122	101	82	225	**4**	425	58
			Lachnospiraceae	unclassified	70179	46	219	444	768	749	594	17	920	654
					70409	28	11	14	116	446	124	85	224	158
					71136	**7**	12	**9**	128	38	55	43	104	17
			Peptococcaceae	Desulfotomaculum	48382	360	70	62	49	18	16	296	192	14
					48537	1190	**1**	**4**	1215	1211	1211	1151	1210	1143
					48912	516	96	157	36	30	29	479	370	**10**
			Ruminococcaceae	Faecalibacterium	24978	271	604	860	934	610	807	**7**	986	846
				unclassified	21981	67	66	28	131	75	42	90	88	15
					22081	48	34	16	**4**	91	71	12	80	92
					27131	315	57	66	110	64	102	227	202	**9**
			Veillonellaceae	unclassified	50276	131	43	20	340	233	240	61	157	769
		MBA08	unclassified	sfA	58422	34	38	105	106	46	43	12	115	42
		SHA-98	unclassified	sfA	58657	371	**9**	31	56	17	135	193	957	798
		Thermoanaerobacterales	Thermoanaerobacteraceae	Caldanaerobacter	55124	1202	1177	1194	120	11	62	1205	473	755
					55641	567	590	732	41	**7**	17	595	349	583
					56021	902	948	967	90	**9**	94	742	817	342
					56022	1163	901	1038	105	**6**	24	1179	376	1058
					56043	1058	1129	1134	154	16	47	1076	1006	728
					56129	1205	1209	1211	183	14	93	1219	628	903
					56298	120	51	85	18	**3**	32	44	83	101
				Moorella	49261	160	14	33	**10**	20	**7**	164	28	458
					49627	387	32	41	**2**	18	35	237	317	389
				Thermacetogenium	55104	1111	964	1005	55	58	**9**	1172	1081	1117
				Thermoanaerobacter	55923	429	672	522	17	13	**1**	129	**1**	221
					55527	435	583	586	85	130	30	149	**9**	242
					55753	1150	1155	1148	339	96	**4**	902	**2**	1123
					55874	222	64	169	61	32	**2**	77	**3**	444
					56277	217	371	203	11	28	**3**	64	**4**	246
				unclassified	55098	101	44	48	64	12	**5**	29	36	144
					55535	781	688	703	40	**5**	**8**	823	**10**	537
					55687	539	140	135	43	15	13	483	406	651
					55976	404	447	581	21	**2**	17	525	92	327
					56212	867	756	876	37	**4**	22	684	290	409
					56249	661	682	795	60	**10**	26	624	410	396
			unclassified	sfD	48502	344	27	22	20	53	90	277	541	768
Gemmatimonadetes	Gemmatimonadetes	unclassified	unclassified	sfB	57790	18	163	273	107	114	65	218	80	250
				sfM	57982	152	25	18	71	29	**6**	118	16	**6**
GN04	MSB-5A5	unclassified	unclassified	sfA	57942	294	**3**	**7**	199	299	89	357	107	103
Lentisphaerae	Lentisphaerae	Z20	unclassified	sfA	54060	17	112	158	253	392	271	499	263	79
OP9	JS1	BA021	unclassified	sfA	53170	317	**2**	**1**	748	589	324	820	398	208
					53788	311	**7**	**6**	311	301	242	197	312	348
		SB-45	unclassified	sfA	52955	527	**8**	206	1142	1005	831	604	1059	970
					53275	116	**6**	**2**	124	84	74	301	179	71
					53682	57	**4**	**8**	118	231	133	11	151	291
Proteobacteria	Alphaproteobacteria	Rhizobiales	Bradyrhizobiaceae	Bradyrhizobium	58984	1094	1065	1085	1121	1043	1045	393	19	1016
					59293	1075	1111	1117	1113	1026	1056	460	18	946
					59986	1044	967	941	1086	526	820	401	17	686
					60019	1133	1203	1190	1191	738	1054	239	12	1112
	Deltaproteobacteria	Desulfovibrionales	Desulfohalobiaceae	Desulfonauticus	57389	977	551	737	782	533	435	819	1042	**1**
			Desulfomicrobiaceae	Desulfomicrobium	56993	16	144	197	22	190	88	23	113	**3**
		Desulfuromonadales	Desulfuromonadaceae	Desulfuromonas	58445	318	158	45	203	379	192	478	396	**8**
		Syntrophobacterales	Desulfobacteraceae	Desulfobacterium	52703	233	28	17	219	405	236	388	623	905
			Syntrophaceae	unclassified	51890	**9**	45	98	350	381	54	**6**	529	170

			Syntrophorhabdaceae	unclassified	51939	304	204	230	321	204	395	451	549	20
	Gammaproteobacteria	Chromatiales	Chromatiaceae	unclassified	2973	619	19	13	228	637	288	551	440	623
		Enterobacteriales	Enterobacteriaceae	Serratia	5959	519	716	745	546	262	67	694	260	16
		Oceanospirillales	Halomonadaceae	Halomonas	56545	1203	1189	1198	245	1206	1100	1192	11	1168
					56972	1049	932	905	16	1004	946	685	222	930
					57013	1184	1196	1163	177	1176	997	1217	**7**	1191
					57181	1148	1148	1183	202	1189	1063	1185	**6**	1150
					57401	1137	1161	1142	130	1183	1123	1096	**5**	1128
					57412	1126	1133	1020	251	1195	1081	1140	15	1145
					57584	1117	946	1027	257	1154	926	920	13	1131
					57608	1129	1128	1081	275	1115	961	965	14	669
					57734	1219	1188	1219	242	1223	1207	1224	20	1217
			Oceanospirillaceae	Marinobacterium	1283	1211	555	1166	**1**	1218	1221	1209	1214	1183
					816	1189	904	1169	**8**	857	967	1181	804	1050
				unclassified	2094	410	244	290	**6**	258	368	597	310	74
			SUP05	unclassified	3429	239	363	461	117	562	565	151	275	11
		Pseudomonadales	Pseudomonadaceae	Pseudomonas	3925	91	275	182	**9**	188	489	675	42	703
					670	15	1135	1124	517	1051	1180	1160	1049	1152
					1960	20	**10**	**3**	**3**	25	19	323	**8**	**5**
					2003	45	113	86	19	31	58	214	111	12
					521	13	140	129	115	120	352	20	606	199
					71	254	456	470	13	222	533	562	206	241
					817	63	296	218	26	125	156	104	124	13
Synergistetes	Synergistia	Synergistales	Synergistaceae	unclassified	56063	117	90	63	214	73	14	99	123	289
			TTA_B6	E6	55392	833	**5**	**5**	97	167	41	944	762	300
Tenericutes	Mollicutes	Mycoplasmatales	Mycoplasmataceae	Ureaplasma	70842	42	52	53	148	145	159	18	154	82
Thermodesulfobacteria	Thermodesulfobacteria	Thermodesulfobacteriales	Thermodesulfobacteriaceae	unclassified	55493	391	162	145	27	95	**10**	298	193	526
					55848	1050	1098	1004	149	**8**	122	1148	913	482
					56099	1216	1217	1220	23	**1**	11	1202	1207	460
Thermotogae	Thermotogae	Thermotogales	Thermotogaceae	Thermotoga	54247	898	717	640	294	96	12	1004	262	217
TM7	TM7-3	EW055	unclassified	sfA	77069	**3**	237	594	971	758	732	480	740	600
WS3	PRR-12	GN03	unclassified	sfC	58579	229	268	334	186	502	359	73	360	19

Table S3. Archaeal taxa comprising the top 10 OTU in each sample from PhyloChip post-scale-normalized hybridization intensity scores. Ranks 1-10 in each sample are bolded and highlighted.

Phylum	Class	Order	Family	Genus	OTU_ID	PC	SB1	SB2	K1	K2	K3
Euryarchaeota	Archaeoglobi	Archaeoglobales	Archaeoglobaceae	Archaeoglobus	74680	**3**	17	15	**1**	**1**	17
	Methanobacteria	Methanobacteriales	Methanobacteriaceae	Methanobacterium	76924	66	64	65	50	65	**4**
				Methanobrevibacter	76097	62	63	64	49	66	**3**
					76442	64	67	67	42	62	**1**
					76902	65	68	69	44	64	**2**
				unclassified	76248	69	69	70	51	74	**5**
					76291	87	88	88	45	86	**6**
					76544	54	71	72	58	60	**8**
	Methanomicrobia	Methanomicrobiales	Methanomicrobiaceae	Methanoculleus	76795	59	**6**	**7**	19	44	72
					76181	17	**3**	**2**	13	35	47
					76345	55	**4**	**5**	11	39	60
					76418	**9**	21	11	27	28	42
					76496	24	**10**	17	31	23	43
					76527	36	**7**	**8**	17	36	65
					76688	11	**10**	**9**	33	27	44
					76690	41	13	**10**	30	42	52
					77013	51	**8**	**6**	**2**	25	58
				unclassified	77135	**7**	28	29	**6**	46	20
			Methanospirillaceae	Methanospirillum	76429	20	**9**	14	22	55	**10**
		Methanosarcinales	Methanosaetaceae	Methanosaeta	76381	85	65	66	**8**	68	84
					76020	70	**1**	**1**	86	87	68
					76865	**8**	23	16	54	14	25
			Methanosarcinaceae	Methanolobus	75957	**1**	47	52	79	54	59
				Methanosarcina	76604	**6**	38	41	47	48	34
				unclassified	77105	**5**	16	26	39	76	23
			unclassified	sfC	76607	**4**	**5**	**4**	23	13	**9**
				sfF	76373	**10**	49	48	66	59	41
	Thermococci	Thermococcales	Thermococcaceae	Pyrococcus	75167	79	84	84	**10**	**6**	13
				Thermococcus	75355	80	80	77	18	**8**	26
					74964	73	74	76	**4**	**2**	11
					75759	77	76	80	**7**	**4**	14
					74633	78	79	81	16	**9**	19
					75086	81	78	78	24	**10**	28
					75334	76	75	74	**5**	**3**	15
					75586	74	77	73	**9**	**5**	16
					75895	83	83	82	12	**7**	21
	Thermoplasmata	E2	pMC2A33	unclassified	76095	31	14	13	**3**	11	29
			WCHD3-02	unclassified	76056	**2**	**2**	**2**	15	38	**7**

Table S4. PhyloChip data summarized for bacteria and archaea commonly found in oil reservoirs. Taxa with the highest ranked OTU hydribization scores also were included (top 20 OTU for bacteria or top 10 OTU for archaea according to relative probe intensity). Data bars represent post-scale-normalized OTU intensity values and are scaled within the group across samples. Taxa within a domain are grouped by the primary functional role commonly associated with a lineage as reported in the petroleum reservoir literature or broader hydrocarbon or marine literature as indicated. Many organisms are metabolically flexible and so potentially participate in multiple pathways, depending on local conditions. Common alternative roles are indicated when known.

Phylum	Class	Order	Family	Genus	Group*	PC	SB1	SB2	K1	K2	K3	SR1	I1	I2	Associated functional role(s)**
p__Euryarchaeota	c__Archaeoglobi	o__Archaeoglobales	f__Archaeoglobaceae	g__Archaeoglobus	1							-	-	-	SP
					2							-	-	-	SP
	c__Thermococci	o__Thermococcales	f__Thermococcaceae	g__Pyrococcus	1							-	-	-	F/SP
				g__Thermococcus	1							-	-	-	F/SP
					2							-	-	-	F/SP
	c__Methanomicrobia	o__Methanosarcinales	f__Methanosaetaceae	g__Methanosaeta	1							-	-	-	M-a
					2							-	-	-	M-a
					3							-	-	-	M-a
			f__Methanosarcinaceae	g__Methanosarcina	1							-	-	-	M-a
				g__unclassified	1							-	-	-	M-a
				g__Methanococcoides	1							-	-	-	M-m
				g__Methanolobus	1							-	-	-	M-m
	c__Methanobacteria	o__Methanobacteriales	f__Methanobacteriaceae	g__Methanobacterium	1							-	-	-	M-h
				g__Methanobrevibacter	1							-	-	-	M-h
				g__unclassified	1							-	-	-	M-h
					2							-	-	-	M-h
	c__Methanomicrobia	o__Methanomicrobiales	f__Methanomicrobiaceae	g__Methanoculleus	1							-	-	-	M-h
				g__Methanoplanus	1							-	-	-	M-h
				g__Methanosphaerula	1							-	-	-	M-h
				g__unclassified	1							-	-	-	M-h
			f__Methanoregulaceae	g__Candidatus Methanoregula	1							-	-	-	M-h
			f__Methanospirillaceae	g__Methanospirillum	1							-	-	-	M-h
	c__Thermoplasmata	o__E2	f__Marine group II	g__unclassified	1							-	-	-	U
			f__pMC2A33	g__unclassified	1							-	-	-	U
			f__WCHD3-02	g__unclassified	1							-	-	-	U
					2							-	-	-	U
		o__Thermoplasmatales	f__Aciduliprofundaceae	g__unclassified	1							-	-	-	U
p__Proteobacteria	c__Gammaproteobacteria	o__Oceanospirillales	f__Oceanospirillaceae	g__Marinobacterium	1										FA
					2										FA
		o__Xanthomonadales	f__Xanthomonadaceae	g__Thermomonas	1										FA
p__Proteobacteria	c__Deltaproteobacteria	o__Myxococcales	f__Myxococcaceae	g__Anaeromyxobacter	1										R/F
p__Firmicutes	c__Bacilli	o__Lactobacillales	f__Lactobacillaceae	g__Lactobacillus	1										F
					2										F
	c__Clostridia	o__Clostridiales	f__Eubacteriaceae	g__Acetobacterium	1										F
					2										F
			f__Lachnospiraceae	g__unclassified	1										F
					2										F
					3										F
			f__Thermoanaerobacterales Family III. Incertae Sedis	g__Thermoanaerobacterium	1										F
		o__Thermoanaerobacterales	f__Thermoanaerobacteraceae	g__Ammonifex	1										F/SP
				g__Caldanaerobacter	1										F
					2										F
					3										F
				g__Moorella	1										F
				g__Thermacetogenium	1										F/Sy/SP
					2										F/Sy/SP
				g__Thermoanaerobacter	1										F/SP
					2										F/SP
				g__unclassified	1										F
					2										F
					3										F
			f__Thermoanaerobacterales	g__Thermovenabulum	1										F/SP/NR/

			Family III. Incertae Sedis			IR/MR
p__Proteobacteria	c__Deltaproteobacteria	o__Desulfuromonadales	f__Pelobacteraceae	g__Pelobacter	1	F
					2	F
	c__Gammaproteobacteria	o__Enterobacteriales	f__Enterobacteriaceae	g__Serratia	1	F
p__Thermotogae	c__Thermotogae	o__Thermotogales	f__Thermotogaceae	g__Kosmotoga	1	F
					2	F
				g__Thermotoga	1	F/SP
					2	F/SP
p__Firmicutes	c__Clostridia	o__Clostridiales	f__Peptococcaceae	g__Pelotomaculum	1	Sy
			f__Syntrophomonadaceae	g__Syntrophomonas	1	Sy
p__Proteobacteria	c__Deltaproteobacteria	o__Syntrophobacterales	f__Syntrophaceae	g__Syntrophus	1	Sy
				g__unclassified	1	Sy
			f__Syntrophobacteraceae	g__unclassified	1	Sy
			f__Syntrophorhabdaceae	g__unclassified	1	Sy
					2	Sy
p__Synergistetes	c__Synergistia	o__Synergistales	f__Anaerobaculaceae	g__unclassified	1	Sy/SP
			f__Synergistaceae	g__Candidatus Tammella	1	Sy
				g__Synergistes	1	Sy
				g__unclassified	1	Sy
				g__vadinCA02	1	Sy
			f__Thermovirgaceae	g__Thermovirga	1	Sy/SP
				g__unclassified	1	Sy
			f__TTA_B6	g__E6	1	Sy
p__Firmicutes	c__Thermolithobacteria	o__Thermolithobacterales	f__Thermolithobacteraceae	g__Thermolithobacter	1	IR
p__Proteobacteria	c__Gammaproteobacteria	o__Alteromonadales	f__Shewanellaceae	g__Shewanella	1	IR
	c__Deltaproteobacteria	o__Desulfuromonadales	f__Geobacteraceae	g__Geobacter	1	IR/SP
p__Aquificae	c__Aquificae	o__Aquificales	f__Aquificaceae	g__Hydrogenobaculum	1	H/SP
			f__Desulfurobacteriaceae	g__unclassified	1	SP
p__Firmicutes	c__Clostridia	o__Clostridiales	f__Peptococcaceae	g__Desulfosporosinus	1	SP
				g__Desulfotomaculum	1	SP/Sy
					2	SP/Sy
			f__Veillonellaceae	g__Desulfosporomusa	1	SP
		o__Desulfitobacterales	f__Desulfitobacteraceae	g__Desulfitobacter	1	SP
		o__Natranaerobiales	f__Anaerobrancaceae	g__Dethiobacter	1	SP
p__Nitrospirae	c__Nitrospira	o__Nitrospirales	f__Thermodesulfovibrionaceae	g__BD2-6	1	SP
p__Proteobacteria	c__Deltaproteobacteria	o__Desulfobacterales	f__Desulfobulbaceae	g__Desulfocapsa	1	SP
				g__unclassified	1	SP
		o__Desulfovibrionales	f__Desulfohalobiaceae	g__Desulfonauticus	1	SP
			f__Desulfomicrobiaceae	g__Desulfomicrobium	1	SP
					2	SP
			f__Desulfovibrionaceae	g__Desulfovibrio	1	SP
		o__Desulfurellales	f__Desulfurellaceae	g__Desulfurella	1	SP
		o__Desulfuromonadales	f__Desulfuromonadaceae	g__Desulfuromonas	1	SP
		o__Syntrophobacterales	f__Desulfobacteraceae	g__Desulfobacterium	1	SP
				g__unclassified	1	SP
			f__Syntrophaceae	g__Desulfobacca	1	SP
p__Thermodesulfobacteria	c__Thermodesulfobacteria	o__Thermodesulfobacteriales	f__Thermodesulfobacteriaceae	g__unclassified	1	SP
					2	SP
					3	SP
p__Proteobacteria	c__Gammaproteobacteria	o__Oceanospirillales	f__Alteromonadaceae	g__Marinobacter	1	H/F/NR
p__Firmicutes	c__Clostridia	o__Halanaerobiales	f__Halanaerobiaceae	g__unclassified	1	H
p__Proteobacteria	c__Gammaproteobacteria	o__Oceanospirillales	f__Halomonadaceae	g__Candidatus Portiera	1	H
				g__Chromohalobacter	1	H
					2	H
				g__Haererehalobacter	1	H
				g__Halomonas	1	H
					2	H
					3	H
p__Acidobacteria	c__Acidobacteria	o__Acidobacteriales	f__Acidobacteriaceae	g__unclassified	1	U
p__Actinobacteria	c__Actinobacteria	o__Actinomycetales	f__Streptosporangiaceae	g__Nonomuraea	1	U
p__Bacteroidetes	c__Bacteroidia	o__Bacteroidales	f__Porphyromonadaceae	g__Dysgonomonas	1	U

			f__RikenellaceaeII	g__unclassified	1	U
					2	U
					3	U
p__Chloroflexi	c__Anaerolineae	o__Anaerolineales	f__Anaerolinaceae	g__A4b	1	U
				g__C1_B004	1	U
					2	U
				g__CFB-26	1	U
				g__GCA004	1	U
				g__H39	1	U
				g__OPB11	1	U
				g__SHD-231	1	U
					2	U
				g__T78	1	U
					2	U
	c__Chloroflexi-4	o__unclassified	f__unclassified	g__sfB	1	U
p__Cyanobacteria	c__Chloroplast	o__Euglenozoa	f__unclassified	g__sfA	1	U
	c__Oscillatoriophycideae	o__Chroococcales	f__Microcystaceae	g__Microcystis	1	U
p__Firmicutes	c__Bacilli	o__Bacillales	f__Bacillaceae	g__Bacillus	1	U
			f__Pasteuriaceae	g__unclassified	1	U
			f__Planococcaceae	g__Planococcus	1	U
		o__Lactobacillales	f__Streptococcaceae	g__Streptococcus	1	U
	c__Clostridia	o__Clostridiales	f__Ruminococcaceae	g__Faecalibacterium	1	U
				g__unclassified	1	U
			f__Sulfobacillaceae	g__YNPFFP6	1	U/SO(?)
			f__Veillonellaceae	g__unclassified	1	U
		o__MBA08	f__unclassified	g__sfA	1	U
		o__SHA-98	f__unclassified	g__sfA	1	U
		o__Thermoanaerobacterales	f__unclassified	g__sfD	1	U
p__Gemmatimonadetes	c__Gemmatimonadetes	o__unclassified	f__unclassified	g__sfB	1	U
				g__sfM	1	U
p__GN04	c__MSB-5A5	o__unclassified	f__unclassified	g__sfA	1	U
p__Lentisphaerae	c__Lentisphaerae	o__Z20	f__unclassified	g__sfA	1	U
p__OP9	c__JS1	o__SB-45	f__unclassified	g__sfA	1	U
					2	U
p__Proteobacteria	c__Alphaproteobacteria	o__Rhizobiales	f__Bradyrhizobiaceae	g__Bradyrhizobium	1	U
					2	U
	c__Deltaproteobacteria	o__Desulfobacterales	f__Nitrospinaceae	g__unclassified	1	U
	c__Gammaproteobacteria	o__Chromatiales	f__Chromatiaceae	g__unclassified	1	U
		o__Oceanospirillales	f__Alteromonadaceae	g__Microbulbifer	1	U
			f__Oceanospirillaceae	g__unclassified	1	U
			f__SUP05	g__unclassified	1	U
		o__Pseudomonadales	f__Pseudomonadaceae	g__Pseudomonas	1	U
					2	U
p__Tenericutes	c__Mollicutes	o__Mycoplasmatales	f__Mycoplasmataceae	g__Ureaplasma	1	U
p__TM7	c__TM7-3	o__EW055	f__unclassified	g__sfA	1	U
p__WS3	c__PRR-12	o__GN03	f__unclassified	g__sfC	1	U
p__WS6	c__SC72	o__unclassified	f__unclassified	g__sfA	1	U

* OTU within genera were grouped according to fold change and then averaged within each group to perserve trends. Fold-change Group 1 (<100), Group 2 (100-500), Group 3 (>500). Fold change was defined by Max/Min across the sample set.

** A = aerobe; F= fermenter; FA=Facultatively anaerobic; H = halophile; IR = iron-reducer; M-a = acetotrophic methanogen; M-h = hydrogenotrophic methanogen; M-m = methylotrophic methanogen; NR = nitrate-reducer; R =may be able to respire many substrates (e.g., U(VI), nitrate, Fe(III)); SO=sulfide-oxidizer; SP = sulfide-producer; Sy = Syntroph; U = undefined for this ecosystem. Note many F/Sy have been shown to degrade hydrocarbons and some Sy also can produce sulfide.
